# Intercellular viral spread and intracellular transposition of *Drosophila* gypsy

**DOI:** 10.1371/journal.pgen.1009535

**Published:** 2021-04-22

**Authors:** Richard M. Keegan, Lillian R. Talbot, Yung-Heng Chang, Michael J. Metzger, Josh Dubnau

**Affiliations:** 1 Program in Neuroscience, Department of Neurobiology and Behavior, Stony Brook University, New York City, New York, United States of America; 2 Medical Scientist Training Program, Department of Neurobiology and Behavior, Stony Brook University, New York City, New York, United States of America; 3 Department of Anesthesiology, Stony Brook School of Medicine, New York City, New York, United States of America; 4 Pacific Northwest Research Institute, Seattle, Washington, United States of America; CNRS: Centre National de la Recherche Scientifique, FRANCE

## Abstract

It has become increasingly clear that retrotransposons (RTEs) are more widely expressed in somatic tissues than previously appreciated. RTE expression has been implicated in a myriad of biological processes ranging from normal development and aging, to age related diseases such as cancer and neurodegeneration. Long Terminal Repeat (LTR)-RTEs are evolutionary ancestors to, and share many features with, exogenous retroviruses. In fact, many organisms contain endogenous retroviruses (ERVs) derived from exogenous retroviruses that integrated into the germ line. These ERVs are inherited in Mendelian fashion like RTEs, and some retain the ability to transmit between cells like viruses, while others develop the ability to act as RTEs. The process of evolutionary transition between LTR-RTE and retroviruses is thought to involve multiple steps by which the element loses or gains the ability to transmit copies between cells versus the ability to replicate intracellularly. But, typically, these two modes of transmission are incompatible because they require assembly in different sub-cellular compartments. Like murine IAP/IAP-E elements, the gypsy family of retroelements in arthropods appear to sit along this evolutionary transition. Indeed, there is some evidence that gypsy may exhibit retroviral properties. Given that gypsy elements have been found to actively mobilize in neurons and glial cells during normal aging and in models of neurodegeneration, this raises the question of whether gypsy replication in somatic cells occurs via intracellular retrotransposition, intercellular viral spread, or some combination of the two. These modes of replication in somatic tissues would have quite different biological implications. Here, we demonstrate that *Drosophila* gypsy is capable of both cell-associated and cell-free viral transmission between cultured S2 cells of somatic origin. Further, we demonstrate that the ability of gypsy to move between cells is dependent upon a functional copy of its viral envelope protein. This argues that the gypsy element has transitioned from an RTE into a functional endogenous retrovirus with the acquisition of its envelope gene. On the other hand, we also find that intracellular retrotransposition of the same genomic copy of gypsy can occur in the absence of the Env protein. Thus, gypsy exhibits both intracellular retrotransposition and intercellular viral transmission as modes of replicating its genome.

## Author summary

The genomes of animals and plants contain a vast quantity of so called “junk DNA” that does not provide obvious function to the organism. But it is increasingly clear that “junk DNA” has more important contributions to both normal function and to dysfunction that can cause disease. A great deal of this “junk” is made up by so called “retrotransposons”, which have many similarities to viruses. Many of them are actually evolutionary relatives of retroviruses, and are able to replicate themselves and insert new copies into the host genome. But unlike retroviruses, which are infectious and replicate by moving from one cell to another, retrotransposons replicate within one cell and re-insert their new copies back into the chromosomes of the cell in which they originated. In this publication, we studied replication of gypsy, which is a well known retrotransposon in fruit flies. We found that gypsy has the ability to replicate within a cell, like a retrotransposon and also has the ability to replicate by moving to a new cell like a retrovirus.

## Introduction

The genomes of plants and animals contain a substantial contribution of sequences derived from transposable elements (TEs). In humans, for example, TE derived sequences represent nearly half of all genetic material [1]. TEs mainly act as selfish genetic elements that replicate within germline tissue, where their de novo inserted copies can be passed to offspring, allowing vertical spread within a population [2,3]. But in the case of the Type I TEs, known as retrotransposons (RTEs), there is now compelling evidence that expression and even replication also occurs in somatic tissues and impacts both normal biology and a variety of age-related diseases [4–7].

Members of the long interspersed nuclear element (LINE), LTR-RTE, and ERV families of RTEs have been found to be actively expressed and even to replicate in somatic tissues, most notably within the nervous system [4,6–25]. Although functional consequences of RTE replication during normal neural development are not established, there is growing evidence that dysfunctional expression has a detrimental impact on organismal fitness during aging [11,16,26–37] and in age-related diseases such as cancer [38–52], autoimmune disorders [53–55] and neurodegenerative disorders such as amyotrophic lateral sclerosis [10,11,56–61], frontotemporal dementia [59], Aicardi-Goutieres syndrome [62,63], Alzheimer’s [64–68], progressive supranuclear palsy [67], multiple sclerosis [69–71], fragile X-associated tremor/ataxia syndrome [72], macular degeneration [73], and Rett syndrome [74].

Like retroviruses, RTEs replicate through an RNA intermediate which is then converted into DNA by an encoded reverse transcriptase enzyme. DNA copies can be inserted into de novo chromosomal sites in the genome, thereby increasing copy number with each successive replication cycle [75–77]. Indeed, a subset of RTEs, the LTR-RTEs, are evolutionarily related to retroviruses. Unlike exogenous retroviruses, both LINE and LTR-RTEs are primarily adapted to make use of an intracellular replication cycle, although there is some evidence for transfer via extracellular vesicles [78–80]. Functional LTR-RTEs encode gag and pol open reading frames, but unlike retroviruses they do not contain an envelope glycoprotein (Env) to mediate inter-cellular spread. Also, they generally target assembly of virus-like particles at the lumen of the ER to facilitate re-entry to the nucleus rather than at the extracellular membrane to facilitate release from the cell [81].

Such LTR-RTEs are believed to be the evolutionary ancestors of exogenous retroviruses, which emerged by a multi-step process that includes the gain of an *Env* gene [82–85] and re-targeting of assembly to the extracellular membrane. This process also has occurred in reverse, leading to ERVs that over time can lose their *Env* gene and re-target their assembly for intracellular replication, acting like LTR-RTEs. Indeed, many genomes contain such ERVs, which straddle the evolutionary transition between LTR-retrotransposon and exogenous retrovirus. Gypsy elements in *Drosophila*, the murine IAP-E elements and the HERV-K elements in human genomes, for example, each retain the viral *Env*, and may therefore have the potential to act as either a virus or a retrotransposon.

Although the bulk of research into somatic retrotransposition has so far focused on LINE elements [5], the gypsy ERV also has been shown capable of replicating in somatic tissues in *Drosophila*, including glial cells, post-mitotic neurons, adipose tissues, and intestinal stem cells [10,11,16,33,58,67,86,87], and HERV-K expression has been detected in ALS patients and in several cancers [57,60,61,88–90]. The expression and replication within somatic tissues of ERVs, which encode functional Env proteins, highlights the importance of understanding their replication cycle. These elements sit on a spectrum between intracellular RTE and extracellular virus. It is not clear whether such elements replicate through intracellular transposition or whether their replication requires them to move genetic material between somatic cells via viral transmission [81,91–97].

We have addressed this question using cultured *Drosophila* S2 cells of macrophage lineage. We used a replication reporter system that we recently developed [11] as well as a series of novel reporters, to test whether or not gypsy replication occurs via intra-cellular transposition or intercellular viral transfer. We find that gypsy can transfer between separate populations of cells in cell culture using both cell-free and cell-associated modes of transmission. We further demonstrate that both forms of transmission between cells requires an intact *Env* open reading frame (ORF). Surprisingly, we also find that in the absence of *Env*, gypsy is able to efficiently complete intracellular retrotransposition.

## Results

### Gypsy-CLEVR and gypsy-mCherry reporters of gypsy replication and expression

**Fig 1:**
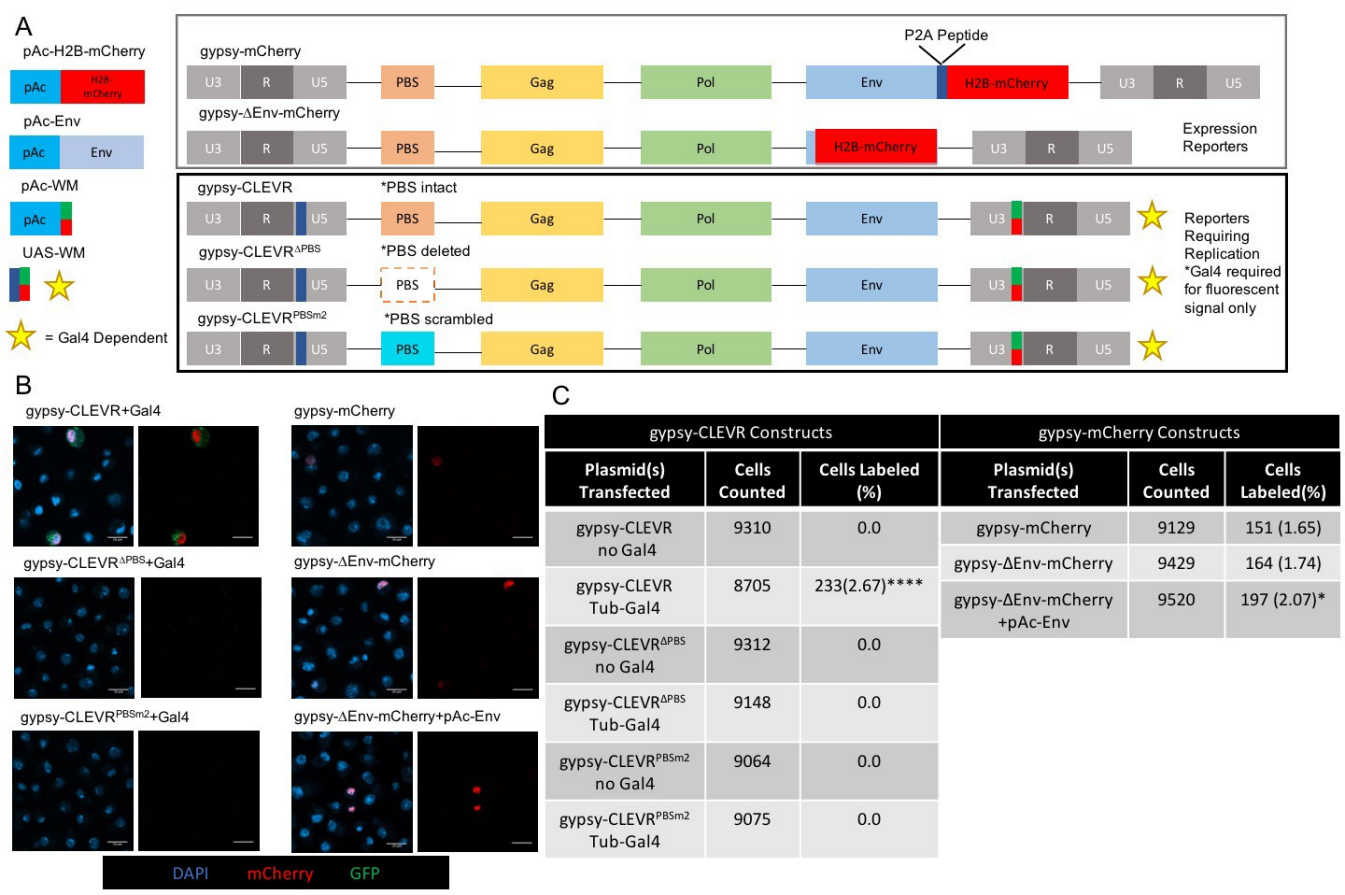
Functional test of reporters marking gypsy replication and expression in Drosophila S2 cells. (A) Cartoon representations of the pAc-H2B-mCherry, pAc-Env, pAc-WM, UAS-WM, gypsy-CLEVR, gypsy-CLEVR^ΔPBS^, gypsy-CLEVR^PBSm2^,(PBS deleted and replaced with a scrambled sequence of the same length (11)) gypsy-mCherry, and gypsy-ΔEnv-mCherry constructs used. The P2A site between Env and mCherry in the gypsy-mCherry construct is denoted in blue. UAS insertion site in gypsy-CLEVR denoted in blue. mCherryP2AmCD8GFP denoted with red/green swatch. Star denotes Gal4 dependence for fluorescence but not replication. (B) Fluorescent images showing WM or mCherry positive S2 cells for gypsy-CLEVR and gypsy-mCherry constructs detected 48 hours after transfection. Scale bars = 10 μm. (C) Quantification showing the percentage of cells labeled for the gypsy-CLEVR constructs with and without Gal4, and the gypsy-mCherry constructs. Quantification is presented as totals cells counted from 3 near equivalent sets of biological replicates. Significance for the gypsy-CLEVR constructs was calculated against gypsy-CLEVR with no Gal4; significance for gypsy-mCherry constructs was calculated against gypsy-mCherry. Significance was determined using the Fisher’s Exact test variant of the Chi^2^ test. Significance values are denoted as: p = <0.05 *, p = <0.001 ***, p = <0.0001**** Raw data for cell counts shown in S1 Table.

We previously described a gypsy reporter system, **C**ellular **L**abeling of **E**ndogenous Retro**v**irus **R**eplication (CLEVR). The gypsy-CLEVR reporter reliably marks cells in which replication of gypsy has occurred and in which a de novo cDNA copy has been reinserted into the genome. This reporter system reliably reports replication of the exogenously supplied gypsy construct both in cell culture and in vivo [10,11]. This gypsy-CLEVR reporter contains the full-length gypsy sequence with a promoterless watermelon (WM) dual fluorescent gene in the 3’LTR and a Gal4-sensitive promoter in the 5’LTR, and it takes advantage of the conserved template switching steps in retrovirus replication to place the Gal4-sensitive promoter upstream to the WM reporter. The gypsy-CLEVR reporter expression requires the replication of gypsy to link the promoter to the reporter and requires the presence of Gal4 to drive the WM signal after replication [11]. The gypsy-CLEVR reporter, and control versions that are unable to replicate due to mutations in the essential primer binding site (PBS) were employed here [11] (Fig 1A). To examine inter-cellular spread of gypsy, we also generated gypsy-mCherry, a more standard reporter of gypsy expression. Gypsy-mCherry relies on the porcine teschovirus-1 2A (P2A) self-cleaving peptide [98] inserted between mCherry and *Env* (Fig 1A) so that the nuclear targeting of mCherry does not interfere with the localization of Env. In contrast with the gypsy-CLEVR reporter, gypsy-mCherry marks any cells in which the construct is activated, differing from CLEVR in that it does not require replication. As the translation of mCherry is linked directly to the *env* encoding (spliced) transcript of gypsy, this reporter is driven by the gypsy-endogenous promoter and does not require Gal4 to display fluorescent signal. We also generated a version of this construct in which the Env protein coding sequence was deleted (Fig 1A).

To confirm the fidelity of the gypsy-CLEVR reporters, *Drosophila* S2 cells were transfected with gypsy-CLEVR and PBS mutant constructs and imaged 48 hours post-transfection (Fig 1A–1C). When co-transfected with tubulin-Gal4, required for the downstream expression of the WM markers, the gypsy-CLEVR reporter showed bright WM fluorescent signal in ~3% of cells (Fig 1B and 1C). In contrast, no labeled cells were detected in the gypsy-CLEVR transfected cells when Gal4 was not present (Fig 1B and 1C). As previously reported [11], deletion or mutation of the primer binding site (gypsy-CLEVR^ΔPBS^, gypsy-CLEVR^PBSm2^) (Fig 1A) eliminated detection of WM labelled cells (Fig 1B and 1C). As controls to ensure a consistent rate of transfection, an actin5c-promoter driven WM dual reporter (pAc-WM), and a Gal4/UAS-driven WM plasmid (UAS-WM) (Fig 1A) were also transfected in parallel. pAc-WM, which does not require Gal4, displayed strong WM signal in ~9% of cells, and UAS-WM labeled 0% and ~10% of cells in the absence and presence of tubulin-Gal4 respectively (S1A and S1B Fig), consistent with our previously reported rates of S2 cells labeled with these constructs [11]. Together, these findings confirm our previous report [11] that gypsy-CLEVR labels S2 cells in which gypsy replication has occurred.

We next tested the gypsy-mCherry and gypsy-mCherry with *Env* deleted (gypsy-ΔEnv-mCherry) constructs to report gypsy expression when transfected into *Drosophila* S2 cells. Both of these constructs produce a nuclear localized mCherry signal when expressed (Fig 1B). We also tested the impact on gypsy-ΔEnv-mCherry when it was co-transfected with a actin5c-driven gypsy-Env plasmid (pAc-Env) expressed in trans (Fig 1B). The gypsy-mCherry, gypsy-ΔEnv-mCherry, and gypsy-ΔEnv-mCherry co-transfected with pAc-Env each labeled ~2% of cells (Fig 1C). For this set of experiments, we used an actin5c-driven mCherry (pAc-H2B-mCherry) as a transfection control. The pAc-H2B-mCherry displayed a strong nuclear mCherry signal in ~4% of cells (S1A and S1B Fig). Therefore, the gypsy-CLEVR and gypsy-mCherry groups of reporter constructs reliably label cells where gypsy has replicated or is expressed respectively, but these experiments do not discriminate between intercellular and intra-cellular replication cycles.

### Cell-associated transmission of gypsy between co-cultured cells

**Fig 2:**
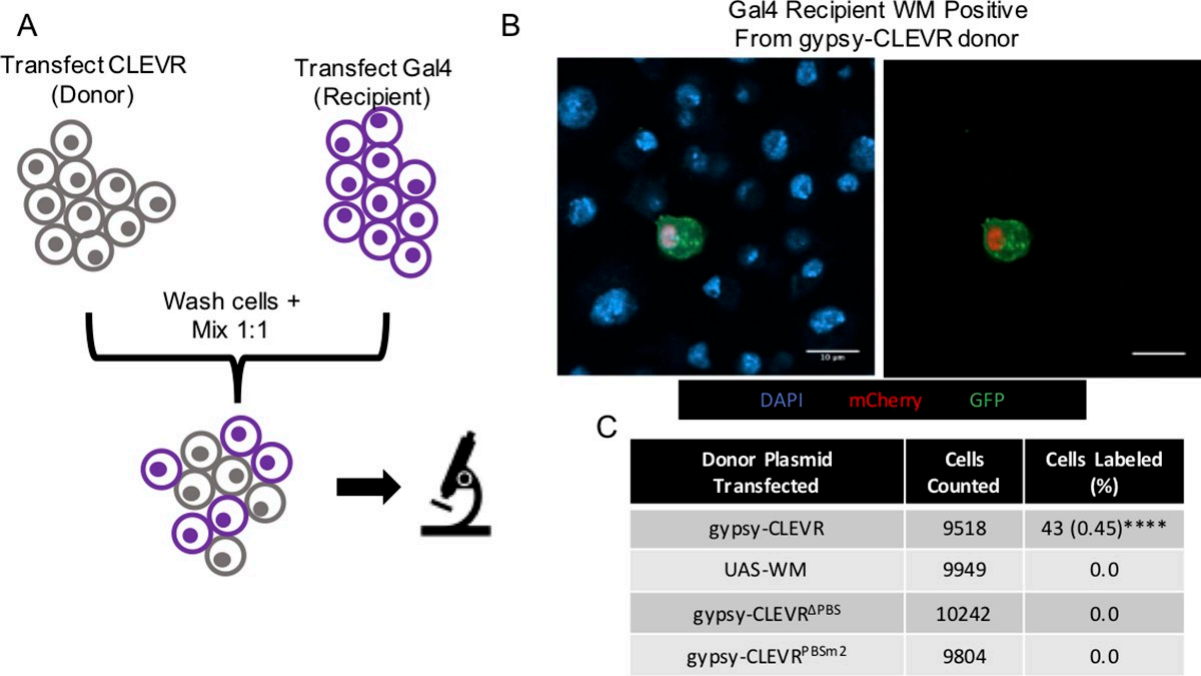
The gypsy-CLEVR reporter reveals that gypsy transfers between cells in contact and integrates into the infected recipient cell. (A) Cartoon schematic showing the experimental design of the co-culture assay. Separate populations of S2 cells are transfected with gypsy-CLEVR or tubulin-Gal4 constructs for 48 hours, washed, and then mixed together in equal proportions for further incubation of 48 hours before imaging. (B) Fluorescent images showing WM labeled cells in the co-cultured gypsy-CLEVR and tubulin-Gal4 cell population. UAS-WM, gypsy-CLEVR^ΔPBS^, and gypsy-CLEVR^PBSm2^ showed no WM labeled cells and are not shown. Scale bars = 10 μm. (C) Quantification showing the percentage of cells expressing the WM reporter for the UAS-WM (control) and gypsy-CLEVR constructs in co-culture with tubulin-Gal4. Quantification is presented as totals cells counted from 3 near equivalent sets of biological replicates. Significance was calculated against UAS-WM. Significance was determined using the Fisher’s Exact test variant of the Chi^2^ test. Significance values are denoted as: p = <0.05 *, p = <0.001 ***, p = <0.0001**** Raw data for cell counts shown in S1 Table.

We next used the gypsy-CLEVR reporter to test whether gypsy is capable of transmitting between cells grown in contact. We took advantage of the Gal4 dependence of the reporter expression in the gypsy-CLEVR construct. The gypsy-CLEVR reporter requires Gal4 to produce a fluorescent signal after replication, but does not require Gal4 for replication. We transfected separate populations of S2 cells with either tubulin-Gal4 or the gypsy-CLEVR reporter. The gypsy-CLEVR transfected populations were designated as “donor cells” while the tubulin-Gal4 transfected populations were designated as “recipient cells”. 48 hours following transfection, cells were washed by centrifugation to remove remaining transfection complex, and then seeded into co-culture at equal ratios (Fig 2A). Cells were then mounted and imaged after 48 hours in co-culture. In this experiment, neither the Gal4 alone nor the gypsy-CLEVR alone is sufficient to yield expression of the dual WM reporter. On the other hand, intercellular transmission of the gypsy-CLEVR followed by integration into the Gal4 expressing recipient cell genome would yield reporter expression. As controls, we also used the gypsy-CLEVR^ΔPBS^ and gypsy-CLEVR^PBSm2^, which possess disrupted primer binding sites (Fig 2A) and therefore can be expressed but cannot replicate. We also used a co-culture control in which one population of cells had been transfected with a Gal4 dependent UAS-WM and the other with the Gal4 itself. The expectation is that there should be no intercellular transmission of the WM transcript when it is not associated with the gypsy-CLEVR construct.

We see clear evidence that gypsy is able to transmit between cells in this cell-associated co-culture assay. When the intact gypsy-CLEVR construct was used, it resulted in positive WM expression detected in ~0.5% of cells, indicating gypsy containing the properly rearranged UAS-WM reporter CLEVR system is capable of moving into tubulin-Gal4 expressing cells (Fig 2B and 2C). In contrast, we observed no WM positive cells when the UAS-WM transfected cells were co-cultured with Gal4 transfected cells, indicating that the reporter cannot move between cells when it is not associated with gypsy. In addition, we observe no WM positive label when gypsy-CLEVR^ΔPBS^ or gypsy-CLEVR^PBSm2^ transfected cells were co-cultured with Gal4 expressing cells (Fig 2C). Thus, gypsy constructs that are unable to generate cDNAs for reinsertion, due to deletion or mutation of the PBS also are unable to report expression in recipient cells grown in contact.

### Cell-free transmission of gypsy

We next tested whether gypsy is capable of cell-free transmission between S2 cells that are not grown in direct cell contact. This assay is conceptually similar to that of the gypsy-CLEVR reporter in co-culture described above. However, in this case, we used a transwell system that utilizes a semi-permeable barrier (0.4 μm) between two separately transfected populations of cells. In a manner similar to that of the co-culture assay, we capitalized on the Gal4 dependence of the WM reporter in the gypsy-CLEVR construct. This construct is capable of replicating independently of Gal4, but cannot express the reporter from the integrated pro-virus unless Gal4 is present. We again separately transfected either the gypsy-CLEVR reporter or Gal4, and we grew these in a transwell cell culture plate to separate the two populations of cells. The culture plates used possess a membrane permeabilized by 0.4 μm pores, which are sufficient to restrict passage of whole cells, the nuclei of which are several microns in diameter, and likely most cellular debris, but would permit transfer of virus particles that likely would be below that size.

**Fig 3:**
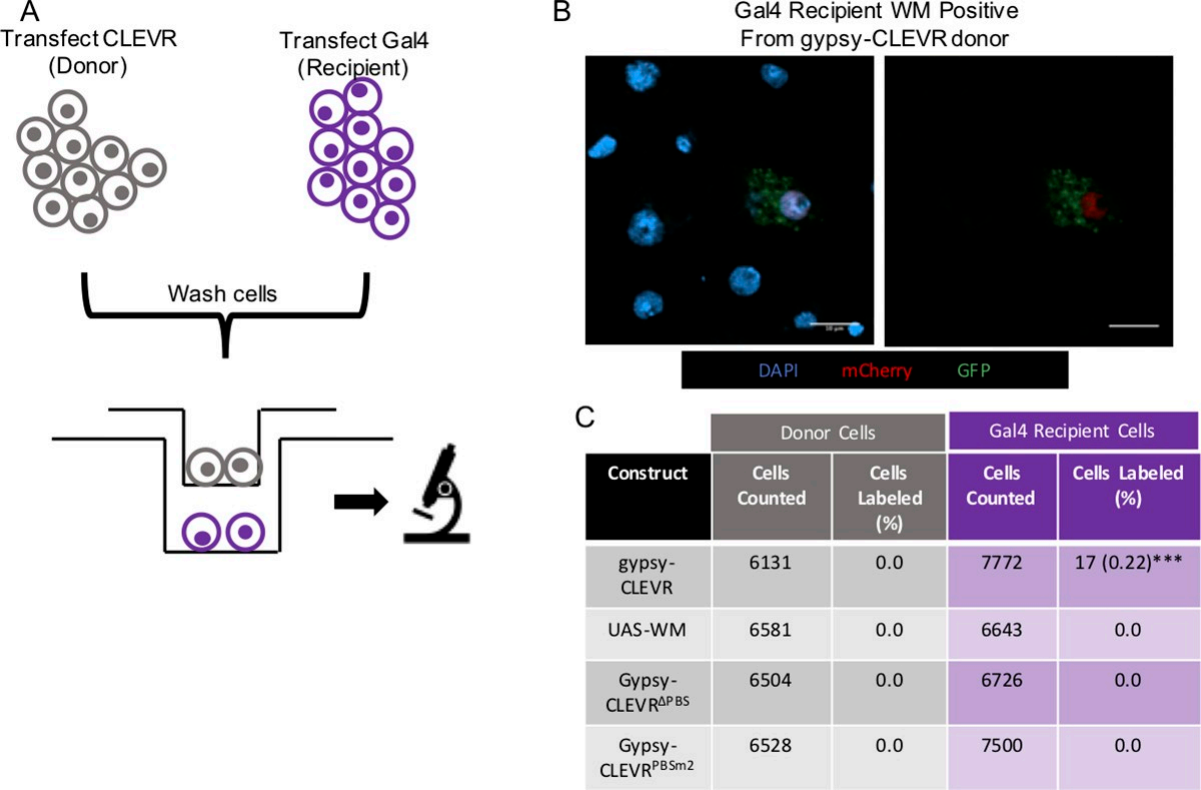
The gypsy-CLEVR reporter reveals intercellular gypsy transmission through a contact restricting membrane. (A) Cartoon schematic showing the experimental design of the transwell assay. Separate populations of S2 cells are transfected with gypsy-CLEVR or tubulin-Gal4 constructs for 48 hours, washed, and then re-seeded on separate sides of a 0.4 μm membrane and further incubated for 48 hours prior to imaging. (B) Fluorescent images showing WM labeled cells in the tubulin-Gal4 cell recipient population. UAS-WM, gypsy-CLEVR^ΔPBS^, and gypsy-CLEVR^PBSm2^ showed no WM labeled cells in the recipient populations and are not shown. No WM labeled cells were detected in the donor populations and are not shown. Scale bars = 10 μm. (C) Quantification showing the percentage of cells expressing the WM reporter for the UAS-WM (control) and gypsy-CLEVR constructs for both the donor and recipient populations in the transwell assay. Quantification is presented as totals cells counted from 3 near equivalent sets of biological replicates. Significance was calculated against UAS-WM. Significance was determined using the Fisher’s Exact test variant of the Chi^2^ test. Significance values are denoted as: p = <0.05 *, p = <0.001 ***, p = <0.0001**** Raw data for cell counts shown in S1 Table.

Here too, we tested transmission of the wild-type gypsy-CLEVR as well as the gypsy-CLEVR^ΔPBS^, and gypsy-CLEVR^PBSm2^, which are unable to replicate due to disruption of the PBS sequences. A separate population of cells was transfected with Gal4 alone. Cells transfected with either the CLEVR constructs or the Gal4 were allowed to incubate on their own for 48 hours, after which the cells were washed by centrifugation and seeded on opposite sides of the membrane in the transwell plate cell-culture dish (Fig 3A). The gypsy-CLEVR transfected populations were designated as “donor cells” while the tubulin-Gal4 transfected populations were designated as “recipient cells”. After an additional incubation of 48 hours in the transwell cell culture plate, both donor and recipient populations were separately mounted and imaged to detect both transfer and directionality of transfer. Expression of the WM reporter was indicative of transfer, as none of the plasmids transfected can produce the WM signal on their own.

Among all the groups, the only population of cells that displayed WM dual fluorescence signal were the “recipient” population of cells expressing tubulin-Gal4 when they were grown on the opposite side of the membrane to the intact gypsy-CLEVR donor population (Fig 3). In this recipient population of cells, ~0.2% of Gal4 transfected cells were found to express the WM reporter (Fig 3B and 3C). No donor populations (gypsy-CLEVR, gypsy-CLEVR^ΔPBS^, gypsy-CLEVR^PBSm2^) or the donor control (UAS-WM) displayed any WM signal, indicating that Gal4 was in no case transferred across the membrane from the recipient to the donor cells (Fig 3C). Further, we did not observe any WM reporter expression in the tubulin-Gal4 recipient populations grown opposite the gypsy-CLEVR^ΔPBS^, gypsy-CLEVR^PBSm2^, that are unable to replicate (Fig 3C). Nor did we observe any expression in the Gal4-expressing recipient cells grown in the transwell below the UAS-WM control donor populations (Fig 3C). In addition to these imaging based findings, we also used PCR to detect gypsy-CLEVR DNA sequences within the recipient cells. To accomplish this, we used several independent PCR strategies. First, we used a nested PCR design that can amplify a product only in the presence of the predicted rearrangement of the LTRs that occurs during replication (S2A Fig). This PCR approach should only amplify from gypsy-CLEVR DNA that has undergone replication through an RNA intermediate. With this approach, we detected a product in cells from either side of the transwell assay. In the recipient cells that are grown opposite to transfected cells, we detected this product from 4 out of 6 independent transwell experiments that utilized the CLEVR construct with an intact PBS and in 0 of 6 lanes with the PBS deleted (S2B Fig). Sanger sequencing of this PCR product confirms that the expected rearrangement has taken place (S2D Fig).

As an independent confirmation that does not rely on nested PCR, we used primers that detect the presence of either the GFP or mCherry reporters. This second set of primers amplify either unreplicated gypsy-CLEVR DNA from the transfected plasmid or cDNA that is produced from replication of the encoded RNA. Because the recipient cells were not transfected with the plasmid, such DNA should only be present if the virus was transferred through the transwell system and was then used to produce a viral DNA. Here too, we detected both GFP and mCherry in the recipient cells from the same 4 out of 6 transwell experiments in which we used the intact gypsy-CLEVR construct and in 0 of 6 wells that used the primer binding site mutant construct (S2C Fig). Together, the imaging and PCR findings demonstrate that gypsy is able to transmit between cells that are not in contact. The fact that such transfer only occurs when the reporter is tethered to an intact gypsy that is able to replicate demonstrates the specificity of this assay. The unidirectional nature of transfer from gypsy-CLEVR expressing cells to tubulin-Gal4 expressing recipient cells, also supports the conclusion that gypsy acts as an infectious retrovirus in cell-culture, capable of cell-free transmission.

### Intercellular transmission of gypsy requires *Env*

Enveloped viruses encode a surface glycoprotein that mediates recognition of cellular receptors and fusion with the cell membrane. Retroviral *Env* genes thus are required both for cell-free and cell-associated transmission. To test whether intercellular transmission of gypsy also is env-dependent, we used the gypsy-ΔEnv-mCherry construct, in which we replaced the gypsy-encoded *env* ORF with that of mCherry. We tested both the gypsy-mCherry with *Env* intact (Fig 1A) and gypsy-ΔEnv-mCherry constructs in the transwell assay that is described above for the gypsy-CLEVR reporter. Unlike the WM reporter in gypsy-CLEVR assay, the expression of mCherry from gypsy-mCherry and gypsy-ΔEnv-mCherry does not require replication of the gypsy RNA genome and does not require co-expression of Gal4.

**Fig 4:**
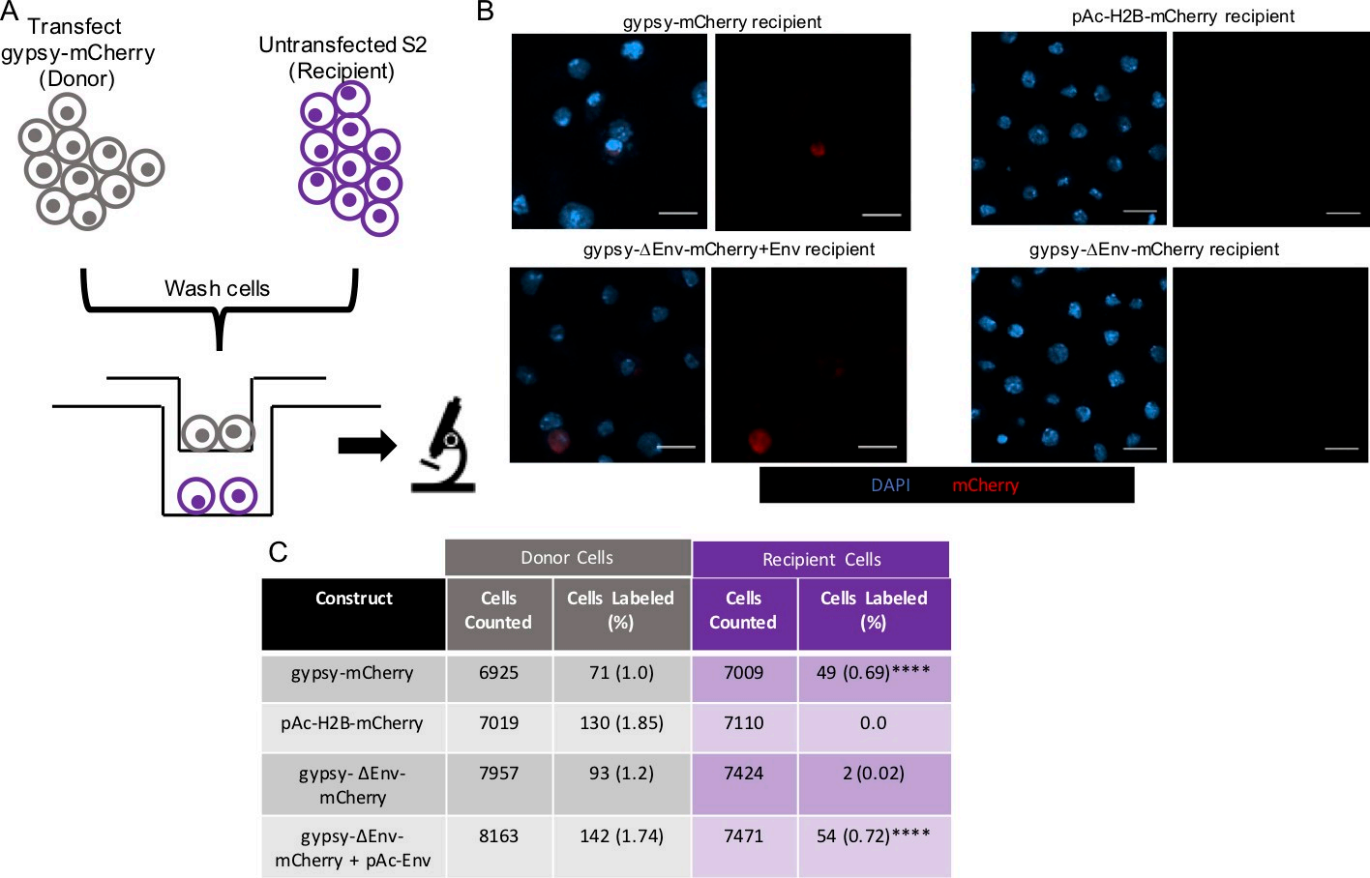
The gypsy-mCherry reporter reveals that intercellular transmission of gypsy requires functional env. A) Cartoon schematic showing the experimental design of the transwell assay. One population is transfected with the gypsy-mCherry constructs for 48 hours, washed, and placed opposite untransfected S2 cells separated by a 0.4 μm membrane for an additional 48 hours prior to imaging. (B) Fluorescent images showing mCherry labeled cells in the S2 cell recipient population. pAc-H2B-mCherry and gypsy-ΔEnv-mCherry recipient populations show no or few labeled cells respectively, and are not shown. Donor populations are not shown. Scale bars = 10 μm. (C) Quantification showing the percentage of cells expressing mCherry for the pAc-H2B-mCherry (control) and gypsy-mCherry constructs for both the donor and recipient populations in the transwell assay. Quantification is presented as totals cells counted from 3 near equivalent sets of biological replicates. Significance was calculated against gypsy-ΔEnv-mCherry. Significance was determined using the Fisher’s Exact test variant of the Chi^2^ test. Significance values are denoted as: p = <0.05 *, p = <0.001 ***, p = <0.0001**** Raw data for cell counts shown in S1 Table.

S2 cells were transfected with either gypsy-mCherry or gypsy-ΔEnv-mCherry. As a further test of the requirement for Env, we also tested whether co-transfection of a pActin-Env (pAc-Env) was able to rescue the env-deficient virus in trans. A pAc-H2B-mCherry plasmid was used as a transfection control. As with the gypsy-CLEVR system described above, transfected cells were first cultured separately for 48 hours (Fig 4A). Following this incubation period, the cells were washed via centrifugation and transferred into the transwell cell culture plate above recipient S2 cells (Fig 4A). Because the gypsy-mCherry constructs do not require presence of Gal4 to visualize reporter expression, the recipient cells used here were untransfected. This offers a numerical advantage over the gypsy-CLEVR reporter in that 100% of the recipient pool of cells are able to report transmission if it occurs. Following a 48-hour incubation in the transwell cell culture plate, both donor and recipient populations of cells were mounted and imaged for expression of nuclear mCherry.

Here, all of the transfected donor population are expected to express nuclear mCherry, and the recipient population of cells would express mCherry if gypsy had transferred across the membrane. Within the donor populations of cells, the control pAc-H2B-mCherry plasmid showed expression that labeled ~2% of cells, reflecting the transfection rate at this time-point (4 days after transfection). The percent of mCherry-expressing donor cells for gypsy-mCherry, gypsy-ΔEnv-mCherry, and gypsy-ΔEnv-mCherry + pAc-Env transfections were ~1%, ~1% and ~2% respectively (Fig 4B and 4C). In the recipient population grown opposite to the control pAc-H2B-mCherry, no cells were found to express the mCherry label, as expected. In contrast, ~0.7% of recipient cells grown opposite to the gypsy-mCherry were found to express the reporter, consistent with the fact that gypsy virus can transmit between cells that are not in contact. But this number dropped to near zero (0.02%) for recipient cells grown opposite to gypsy-ΔEnv-mCherry transfected donor cells. This strongly supports the conclusion that the gypsy *Env* gene is required for transmission. This deficiency in intercellular transmission with the *Env* deleted construct also could be rescued when Env was expressed in trans. When the gypsy-ΔEnv-mCherry construct was co-transfected with pAc-Env, ~0.7% of the recipient cells expressed mCherry (Fig 4B and 4C). Together, these results confirm that gypsy is capable of cell-free transmission, but also show that this transmission is reliant upon the presence of functional *Env*.

### Intracellular transposition of gypsy is *Env* independent

The above findings indicate that gypsy retains the ability to transmit between cells under both cell-associated and cell-free conditions, and such transmission is *Env* dependent. Unlike retroviruses, LTR-retrotransposons typically utilize an intracellular replication cycle that is not env-dependent, but intracellular replication also requires significant differences in targeting within the cell. The *Env* dependent inter-cellular transmission of gypsy would necessitate assembly at the extracellular membrane. We wondered therefore if gypsy, which classically has been thought of as a retrotransposon, is even capable of replicating intracellularly. To test this, we generated a gypsy-CLEVR construct in which we had introduced a frameshift mutation within the *Env* ORF (S3 Fig). Because the gypsy-CLEVR reporter labels cells only after reverse transcription and template switching [10,11], this reporter provides a means to distinguish replication events from mere expression. Because expression of the WM dual reporter that is contained on the gypsy-CLEVR construct is Gal4 dependent, we co-transfected with a Gal4 expression construct.

**Fig 5:**
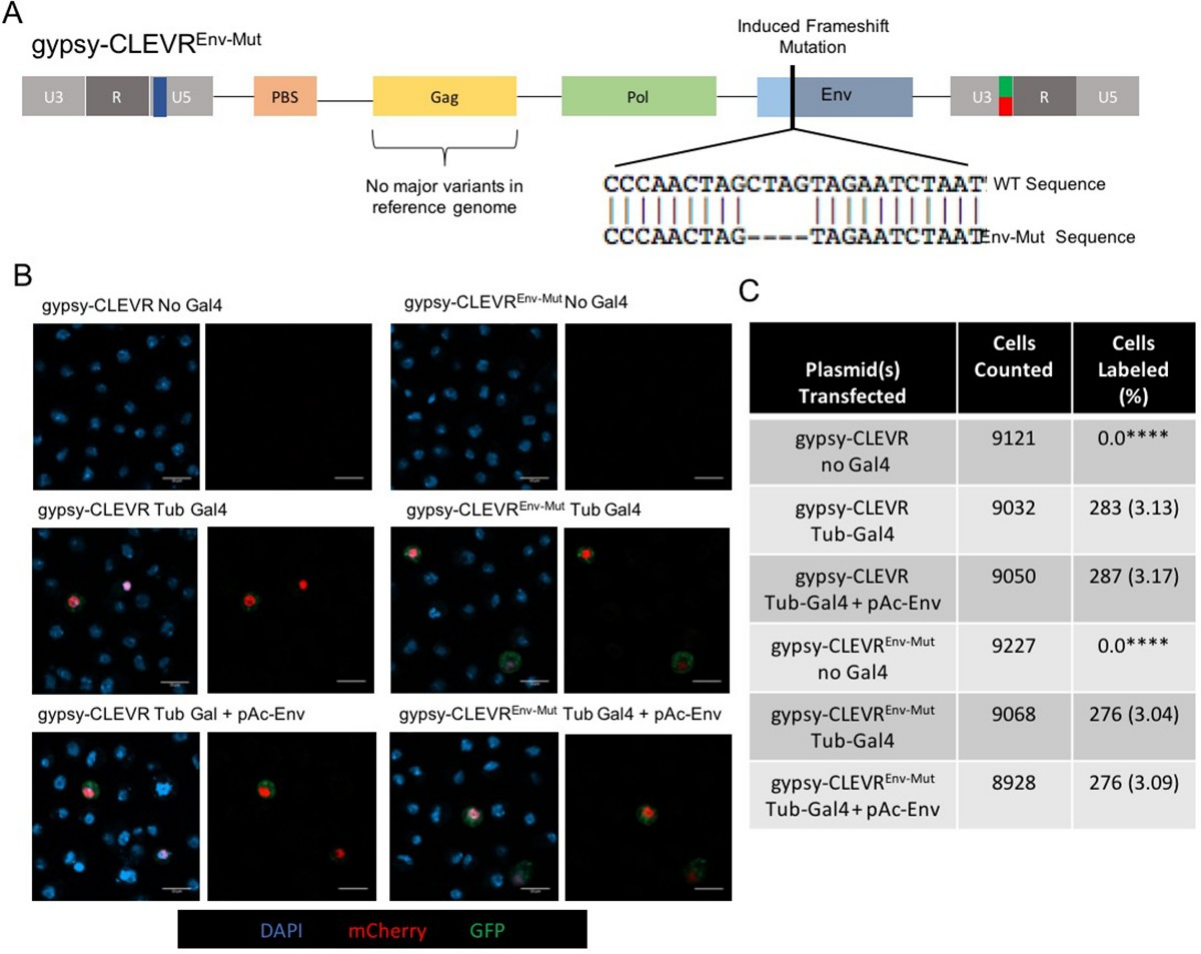
Functional env is not required for the intracellular retrotransposition of gypsy. Cartoon schematic showing the overall structure of the gypsy-CLEVR^Env_mut^ construct, which is identical to gypsy-CLEVR but has a frameshift mutation within the env ORF, seen in detail in the sequence comparison directly below. B) Fluorescent images showing the absence of WM signal in gypsy-CLEVR and gypsy-CLEVR^Env_mut^ populations lacking Gal4, and positive WM signal in gypsy-CLEVR and gypsy-CLEVR^ΔEnv^ when co-transfected with tubulin Gal4 as well as with pAc-Env. C) Quantification of the percentage of cells that showed positive WM signal for gypsy-CLEVR and gypsy-CLEVR^ΔEnv^ with and without Gal4, as well as with pAc-Env. No statistically significant differences were found absent gypsy-CLEVR and gypsy-CLEVR^Env_mut^ lacking the presence of Gal4. Quantification is presented as totals cells counted from 3 near equivalent sets of biological replicates. Significance was determined using the Fisher’s Exact test variant of the Chi^2^ test. Significance values are denoted as: p = <0.05 *, p = <0.001 ***, p = <0.0001**** Raw data for cell counts shown in S1 Table.

S2 cells were transfected with tubulin-Gal4 as well as either gypsy-CLEVR or gypsy-CLEVR^Env_mut^. Each of the above two constructs were tested both with and without pAc-Env to provide Env expression in trans (Fig 5A). After transfection, the populations of cells were incubated for 48 hours, mounted and imaged to detect the presence of the WM reporter. When co-transfected with tubulin-Gal4, the gypsy-CLEVR plasmid produced strong WM label in 3.2% of cells imaged. This is consistent with the robust levels of gypsy replication in S2 cells that we have observed previously [11]. When this gypsy-CLEVR construct was co transfected with both tubulin-Gal4 and an additional source of pAc-Env expressed in trans, the fraction of labeled cells remained at 3.2% (Fig 5B and 5C). Thus, Env levels are not limiting the rate of replication of the gypsy-CLEVR construct. When gypsy-CLEVR^Env_mut^ was co-transfected with tubulin-Gal4, 3.0% of cells were WM labeled, a rate that is statistically indistinguishable from that of the intact gypsy-CLEVR construct. Similarly, when the gypsy-CLEVR^Env_mut^ was co-transfected with both tubulin-Gal4 and pAc-Env, 3.1% of cells were labeled with WM (Fig 5B and 5C). Here too, the rate of replication of gypsy-CLEVR^Env_mut^ is indistinguishable from that of the intact gypsy-CLEVR irrespective of whether an additional source of Env is supplied in trans. Taken together, these findings demonstrate that gypsy is capable of intracellular retrotransposition that is independent of Env.

## Discussion

ERVs can defy a clear definition, as some act as retroviruses and others act as LTR-RTEs, leaving these elements in a sort of evolutionary “gray area”. From an evolutionary perspective, it is thought that LTR-RTEs are the likely ancestors of retroviruses, and all vertebrate retroviruses come from a single lineage [85,99]. The emergence of retroviruses is thought to have involved a multi-step process that includes targeting to the cell membrane and the incorporation of a surface glycoprotein (Env). This process also has likely occurred in reverse, as some ERVs have lost their Env and developed the ability to re-target internally, and in some cases, these have even been called RTEs, despite the different evolutionary history. Indeed, the fact that the invertebrate gypsy element contains an *env* gene is suggestive that the gypsy element is capturing the process of the generation of a new lineage of retrovirus from RTE ancestors.

Here, we demonstrate that gypsy, one of the most well-known LTR-RTEs, is able to replicate intracellularly as an RTE, but also can transmit between cells grown in culture as a virus. This intercellular transmission can occur both for cells grown in close contact and by a cell free mechanism. We observe such intercellular movement with two different reporters, one of which labels any recipient cells that express gypsy encoded proteins and the other of which only labels recipient cells that have a gypsy provirus which has gone through reverse transcription. With this second reporter, we can only label the infected recipient cells when the PBS, which is essential for viral replication, is intact. Finally, we demonstrate that intercellular transmission of gypsy occurs by a mechanism that requires functional *Env*, consistent with the idea that transmission occurs via a viral mechanism. It is worth noting that it has previously been suggested that a tagged gypsy element may be capable of transmission in vivo from somatic follicle cells to the oocyte, and that this may occur in the absence of a functional *Env* [100]. Although we do not observe *Env* independent intercellular transmission in S2 cells, we cannot rule out the possibility that in some contexts, gypsy exhibits a third mode of replication that is *Env* independent but intercellular.

The gypsy element, which has been termed an errantivirus, has long been thought to possess features of an infectious retrovirus [92,101], as also is thought to be true for the *Drosophila* ZAM element (e.g. [102]). Pseudotyping of Moloney murine leukemia virus with gypsy env is sufficient to confer entry to insect cells [103], which demonstrates that the gypsy envelope glycoprotein is functional. Several reports document that virus-like particles are present in *Drosophila* ovaries from genotypes in which gypsy replication is taking place [94,104]. More striking is the observation that horizontal transmission of gypsy can occur when larvae from strains that have no functional gypsy elements are fed extracts from ovaries of animals with active gypsy [91,94]. The experiments that we describe here demonstrate that gypsy indeed possesses qualities of a retrovirus, enabling Env-dependent infectious transmission. More surprisingly, gypsy also can replicate just as efficiently as an intracellular RTE in the absence of Env. Given the complex functional changes that underlie evolutionary transition between LTR-RTEs and retroviruses, this dual mode of replication is unexpected. This point is driven home by a comparison to the murine intracisternal A-type particle (IAP) and the related intracisternal A-type particle with Env (IAP-E).

The IAP elements, which are murine ERVs that lack env, follow a purely intracellular RTE-like replication lifecycle, remaining within the cell where they are targeted to the lumen of the endoplasmic reticulum, which is contiguous with the perinuclear space [81]. Conversely, the mouse IAP-E element, which possesses a functional env ORF and is therefore more closely related to the ancestral exogenous virus that gave rise to all IAP and IAP-E ERVs, has been shown to replicate following an intercellular lifecycle, producing exogenous virus that buds at the membrane and infects neighboring cells [81]. Although the loss of *Env* is important in the evolutionary transition from the viral life cycle into to an RTE-like lifecycle, mouse IAP and IAP-E elements also differ in the gag ORF, where amino acid variation within the gag proteins of these elements are sufficient to change the targeting to be compatible either with intercellular or intracellular replication [81]. Strikingly, hybrid IAP-E elements in which the N-terminal region of gag is substituted from IAP are unable to produce viral particles at the membrane because of mis-targeting of gag.

The situation with gypsy appears to be quite different from that of IAP-E. Unlike these murine elements, all of the intact gypsy copies that are identified in the *Drosophila* reference genome appear to contain an *Env* reading frame. And we see no evidence for existence of gypsy variants with significant substitutions in gag that might provide for two classes of element as is the case with IAP/IAP-E. Moreover, in contrast with IAP/IAP-E, the specific variant of gypsy that we used to construct our reporters appears capable of both modes of replication. The ability of ERVs to replicate via intracellular vs intercellular mechanisms may have significant biological impact.

Expression and replication of RTEs and ERVs have been found in somatic tissues both during normal development [5,7,8,11–13,16,17,19,21–24,83,105,106], in advanced aging [11,16,27,28,33,34,86,107–109] and in diseases of aging such as neurodegeneration [10,57–61,65,67,72,96,110–112], and cancer [9,48–52,113–120]. The functional consequences of somatic expression and replication of RTEs/ERVs are only beginning to be understood, and it is not known if inter-cellular transmission occurs in vivo. But there already is evidence that cells that exhibit RTE/ERV replication may have non-cell autonomous impacts on surrounding tissue [10,28]. It now is established that HERV-K [96,97], IAP-E [81,95] and gypsy each are functional viruses in cell culture and IAP [81] and gypsy have intracellular replication cycles as well. While LINE elements do not encode machinery for viral transmission, there is recent evidence that human-specific LINE-1 elements can transmit between cells in culture via extracellular vesicles [79]. In addition, the Arc genes in both mammals and in *Drosophila* have recently been found to have their ancestral origin from a gypsy-family gag protein, and Arc has been shown to bind and transport mRNA cargo between neurons [78,80]. Together, these findings reveal the dual replication strategies used by an element in transition between a retrotransposon and a virus and raise the possibility that ERVs and RTEs may provide routes for transfer of genetic information between cells within an organism.

## Materials and methods

### Constructs

To generate pAc-Env, the Env was amplified from the gypsy-CLEVR plasmid using polymerase chain reaction (PCR) and was inserted into the multiple cloning site (MCS) of the pAc5.1 C vector (Thermo Fisher Scientific) with a NotI and KpnI digestion. The gypsy-CLEVR^Env_mut^ was constructed by digesting the gypsy-CLEVR plasmid with BcuI, ethanol precipitated, and treated with Klenow before ligation, resulting in a frame shift occurring within the Env of gypsy-CLEVR at position 13,847 in the CLEVR reporter. Gypsy Env is located between 13,470–14,916 within the CLEVR construct. To generate the S2 cell-based reporter pAc-H2B-mCherry, the nuclear localization reporter H2B-mCherry-HA was amplified from Watermelon (WM) reporter described in our previous study [11] by PCR. The PCR-amplified H2B-mCherry-HA was then inserted into the XhoI site of the *Drosophila* constitutive expression vector, pAc5.1/V5-His version C (V411020, Thermo Fisher Scientific). In order to test the transferring ability of gypsy, the gypsy backbone used in previous publication [11] was amplified and cloned into the NotI/XbaI sites of pAc5.1/V5-His version B (V411020, Thermo Fisher Scientific). To synthesize the final gypsy-H2B-mCherry vector, the gypsy backbone was first digested with XhoI which flanks the 6.7 kb fragment with all three ORFs. This was cloned into the XhoI site of N1-EGFP vector (N1-gypsy-XhoI). The H2B-mCherry-HA DNA fragment from WM was then added the DNA sequences between AseI site and the end of gypsy ORF3 (env) via a PCR designed to include the P2A linking peptide sequences but with the stop codon of gypsy ORF3 (env) removed. The junction of the end of gypsy ORF3 (env) through the 3’ end at the XhoI site was PCR amplified and fused to the end of the AseI-ORF3-P2A-H2B-mCherry fragment with an engineered BamHI site. The sequence between AseI-BamHI of N1-gypsy-XhoI was then replaced by this final AseI-ORF3-P2A-H2B-mCherry-BamHI fragment (N1-gypsy-H2B-mCherry). The DNA sequence between 2 XhoI sites of N1-gypsy-H2B-mCherry, with the three ORFs and H2B-mCherry, was then moved to replace the DNA fragment between 2 XhoI sites of pAc-gypsy to generate the final gypsy-H2B-mCherry. To synthesize pAc-gypsy-H2B-mCherry^ΔEnv^, the whole gypsy ORF3 (env) was deleted from gypsy backbone and replaced with an H2B-mCherry-HA fragment, but the initial AG**G**TTCACCCTCATG nucleotides from env were maintained in order to provide the endogenous splicing accepting site to receive the alternative splicing stat codon **AT**GT from gypsy ORF1 (gag) [93].

### Cell culture

*Drosophila* S2 cells (R69007, Thermo Fisher Scientific) were cultured in Schneider’s Drosophila Media (Thermo Fisher Scientific) supplemented with 10% Fetal Bovine Serum (Thermo Fisher Scientific) and Pennicillin-Streptomycin-Glutamine (Thermo Fisher Scientific), in 75cm^2^ flasks (Flask info). Cells were transfected with 1.5ug of each plasmid DNA with the Effectene transfection kit (Qiagen). After 48 hours in transfection complex, cells were fixed in 4% Paraformaldehyde and mounted on coverslips coated in 0.5mg/ml Concanavalin A and ProLong Diamond Antifade Mountant with DAPI (Thermo Fisher Scientific). All images were taken on a Zeiss Confocal microscope and quantified under blinded conditions using the cell counter feature in FIJI.

### Co-culture

Prior to co-culture, cells were transfected with individual plasmids and incubated for 48 hours in transfection complex. Following this incubation, cells were washed 3 times with 5ml of Schneider’s Drosophila Media and co-cultured at a 50:50 ratio in 75cm^2^ flasks. After 48 hours in the co-culture condition, cells were fixed in 4% Paraformaldehyde and mounted on coverslips coated in 0.5mg/ml Concanavalin A and ProLong Diamond Antifade Mountant with DAPI.

### Transwell

Prior to introduction into the Transwell system, cells were transfected with individual plasmids and incubated for 48 hours in transfection complex. Following this incubation, cells were washed 3 times with 5ml of Schneider’s Drosophila Media and recipient and donor cells were moved to opposing sides of a 6-well, 0.4um Transwell plate. Following 48 hours in the Transwell plate, cells from both sides of the plate were individually fixed in 4% Paraformaldehyde and mounted on coverslips coated in 0.5mg/ml Concanavalin A and ProLong Diamond Antifade Mountant with DAPI.

### Statistical analysis

All data was analyzed using the Chi^2^ with Yate’s correction analysis in order to obtain a P value for significance between separate groups. For comparisons incorporating multiple zeroes, the Fisher’s Exact test variant of the Chi^2^ test was used. Significance values are denoted as: p = <0.05 *, p = <0.01**, p = <0.001 ***, p = <0.0001****

### PCR primers used

The following primers were used to amplify gypsy Env from the gypsy-CLEVR construct:

- F: GGTACCCAAAACATGatGTTCACCCTCATGATGTTCATACC
- R: GGGAGTAGTTAACAACTAAGCGGCCGCAATTTAGCGCGC

Reverse complement of the R primer:

- GCGCGCTAAATTGCGGCCGCTTAGTTGTTAACTACTCCC

Primers used to detect gypsy-CLEVR that was transferred in the trans-well assay:

- 1R-F 5’-ACAATGTATTGCTTCGTAGC-3’
- 1R-R 5’-AACTACCCTGTTTGTCGCCT-3’
- 2R-F 5’-CTATTTATACTCCGGCGCTC-3’
- 2R-R 5’-CGGAGTACTGTCCTCCGAGC-3’
- GFP-F 5’-ACTTTTTCAAGTCGGCGATG-3’
- GFP-R 5’-CACGGAACCGTCCTCTATGT-3’
- mCherry-F 5’-CCTGTCCCCTCAGTTCATGT-3’
- mCherry-R 5’-CTTCAGCTTCAGCCTCTGCT-3’

### Genomic PCR, genomic nested PCR and sequencing

Genomic DNA was extracted from S2 cells transfected with gypsy-CLEVR and gypsy-CLEVRΔ^PBS^ by PureLink Genomic DNA Kit (Thermo Fisher Scientific). The extracted genomic DNA was followed by two rounds of standard PCR in a nested fashion. Primer 1R-F and primer 1R-R were used in the first round PCR and the product of the predicted size from first round PCR (Red Box in S2B Fig) was extracted from the gel and amplified by primer 2R-F and primer 2R-R. The predicted size range of the PCR product (black arrowhead in S2B Fig) from the second round of PCR was extracted from the gel for sequencing. The same DNA samples were also tested by standard PCR using primer GFP-F and primer GFP-R, or mCherry, mCherry-F and mCherry-R.

## Supporting information

S1 Fig Control plasmids function in *Drosophila* S2 cell culture. A) Fluorescent images showing mCherry labeled nuclei present in approximately 4% of pAc-H2B-mCherry transfected cells, as well as WM signal expressed in approximately 9.6% and 10.3% of pAc-WM and UAS-WM cotransfected with Tub Gal4 transfected cells respectively. UAS-WM, when not cotransfected with a Gal4 plasmid showed no expression of the WM reporter. B) Quantification of the cells counted and the percentage and number (in parentheses) of cells expressing the control pAc-H2B-mCherry, pAc-WM, and UAS-WM with and without Gal4 plasmids. Quantification is presented as totals cells counted from 3 near equivalent sets of biological replicates.
(TIFF)

S2 Fig Gypsy-CLEVR viral transfer detected by PCR of recipient cells. *Drosophila* S2 cells transfected with Gypsy-CLEVR (sending cells) were grown in a trans-well apparatus opposite to untransfected S2 (recipient) cells. The PBS deletion mutant of this construct was used as a control that can become expressed but cannot be used to generate cDNA. DNA was isolated from recipient cells and gypsy-CLEVR DNA was detected by PCR using several different primer designs. First, a nested PCR scheme (**A**) was used to selectively amplify gypsy-CLEVR sequences that had undergone the predicted rearrangement that is associated with replication. The first round PCR primers (1R-F and 1R-R) were used to enrich for a 5’ fragment of gypsy elements that included the 5’LTR and internal sequences including the PBS. Products of this reaction (**B, left panel**) include endogenous gypsy sequences (bright band at ~500nt) as well as a predicted larger fragment derived from the gypsy-CLEVR construct if present. Although not visible on this gel, DNA from the predicted size region (Red rectangle) was isolated and used as template for a second round of PCR using primers that are specific to the HSP70-TATA sequences of the WM reporter (2R-F) and the UAS region (2R-R). This second PCR can only amplify from template that has undergone replication, leading to rearrangement, placing the WM sequence into the 5’-LTR, nearby to the UAS-sequences. This reaction results in amplification of a product of the predicted size (**B, right panel**) when the wild-type PBS construct is used, but not when using the construct with the PBS mutation. Two independent experiments (Batch 1, B1; Batch 2, B2) yielded similar results. Batch 1 and 2 each consisted of 3 independent trans-well cultures with each construct, and this PCR product was detected in 4 of those 6 experiments with the intact PBS construct and 0 of 6 with the PBS mutant (**panel B, right and not shown**). We also detect both the GFP and mCherry fluorescent reporters (**C**) using primers specific to those sequences. Amplification of these products does not require the rearrangement that is associated with replication, but their presence in untransfected recipient cells is indicative of viral transfer. Sanger sequencing (**D**) confirms that the PCR product from nested PCR (**B**) has identical sequence and the expected rearrangement when it is detected in either the recipient or sending cells. We note the presence (Red “G”) of a single-nucleotide polymorphism in the LTR that is unique to the transgenic gypsy-CLEVR construct relative to the endogenous elements in the genome.
(TIF)

S3 Fig Sequence analysis of gypsy-CLEVREnvmutation. A) Nucleotide sequence of gypsy Env. B) Amino Acid sequence of gypsy Env. C) Clustal analysis comparing WT gypsy Env to gypsy-CLEVR^Env_mut^.Highlighted portions of the sequence represent the following: Yellow- Signal Peptide, Teal- Surface Protein Domain, Red- Induced Point Mutation, Green- Protein Cleavage Site, Light Blue- Transmembrane Region.
(TIFF)

S1 Table Raw Data for All figures. S1 Table contains the raw data for all experimental replicates performed in Figs 1–5. Table A,B,C,D and E in S1 Table contain the raw data for all experimental replicates performed in Figs 1–5 respectively. Column 1 in each table designates the constructs transfected; numbers after the construct name in each row of column 1 correspond to each image captured on one slide (25 total) for each biological replicate. Columns 2 to 3 correspond to total cells counted and labeled cells counted respectively within one biological replicate. Columns 4 to 5 and 6 to 7 are identical but for the second and third biological replicates.
(XLSX)

## References

[1] Huang CR, Burns KH, Boeke JD. Active transposition in genomes. Annu Rev Genet. 2012;46:651–75. 10.1146/annurev-genet-110711-155616 23145912 PMC3612533

[2] Kazazian HH Jr. Mobile elements: drivers of genome evolution. Science. 2004;303(5664):1626–32. 10.1126/science.1089670 15016989

[3] Babushok DV, Kazazian HH Jr. Progress in understanding the biology of the human mutagen LINE-1. Hum Mutat. 2007;28(6):527–39. 10.1002/humu.20486 17309057

[4] Dubnau J. The Retrotransposon storm and the dangers of a Collyer’s genome. Curr Opin Genet Dev. 2018;49:95–105. 10.1016/j.gde.2018.04.004 29705598 PMC5975205

[5] Faulkner GJ, Billon V. L1 retrotransposition in the soma: a field jumping ahead. Mob DNA. 2018;9:22. 10.1186/s13100-018-0128-1 30002735 PMC6035798

[6] Reilly MT, Faulkner GJ, Dubnau J, Ponomarev I, Gage FH. The role of transposable elements in health and diseases of the central nervous system. J Neurosci. 2013;33(45):17577–86. 10.1523/JNEUROSCI.3369-13.2013 24198348 PMC3818539

[7] Richardson SR, Morell S, Faulkner GJ. L1 retrotransposons and somatic mosaicism in the brain. Annu Rev Genet. 2014;48:1–27. 10.1146/annurev-genet-120213-092412 25036377

[8] Baillie JK, Barnett MW, Upton KR, Gerhardt DJ, Richmond TA, De Sapio F, et al. Somatic retrotransposition alters the genetic landscape of the human brain. Nature. 2011;479(7374):534–7. 10.1038/nature10531 22037309 PMC3224101

[9] Burns KH. Transposable elements in cancer. Nat Rev Cancer. 2017;17(7):415–24. 10.1038/nrc.2017.35 28642606

[10] Chang YH, Dubnau J. The Gypsy Endogenous Retrovirus Drives Non-Cell-Autonomous Propagation in a Drosophila TDP-43 Model of Neurodegeneration. Curr Biol. 2019;29(19):3135–52 e4. 10.1016/j.cub.2019.07.071 31495585 PMC6783360

[11] Chang YH, Keegan RM, Prazak L, Dubnau J. Cellular labeling of endogenous retrovirus replication (CLEVR) reveals de novo insertions of the gypsy retrotransposable element in cell culture and in both neurons and glial cells of aging fruit flies. PLoS Biol. 2019;17(5):e3000278. 10.1371/journal.pbio.3000278 31095565 PMC6541305

[12] Coufal NG, Garcia-Perez JL, Peng GE, Yeo GW, Mu Y, Lovci MT, et al. L1 retrotransposition in human neural progenitor cells. Nature. 2009;460(7259):1127–31. 10.1038/nature08248 19657334 PMC2909034

[13] Eickbush MT, Eickbush TH. Retrotransposition of R2 elements in somatic nuclei during the early development of Drosophila. Mob DNA. 2011;2(1):11. 10.1186/1759-8753-2-11 21958913 PMC3190326

[14] Kazazian HH Jr., Moran JV. Mobile DNA in Health and Disease. N Engl J Med. 2017;377(4):361–70. 10.1056/NEJMra1510092 28745987 PMC5980640

[15] Kubo S, Seleme MC, Soifer HS, Perez JL, Moran JV, Kazazian HH Jr., et al. L1 retrotransposition in nondividing and primary human somatic cells. Proc Natl Acad Sci U S A. 2006;103(21):8036–41. 10.1073/pnas.0601954103 16698926 PMC1472425

[16] Li W, Prazak L, Chatterjee N, Gruninger S, Krug L, Theodorou D, et al. Activation of transposable elements during aging and neuronal decline in Drosophila. Nat Neurosci. 2013;16(5):529–31. 10.1038/nn.3368 23563579 PMC3821974

[17] Muotri AR, Chu VT, Marchetto MC, Deng W, Moran JV, Gage FH. Somatic mosaicism in neuronal precursor cells mediated by L1 retrotransposition. Nature. 2005;435(7044):903–10. 10.1038/nature03663 15959507

[18] Bae BI, Jayaraman D, Walsh CA. Genetic changes shaping the human brain. Dev Cell. 2015;32(4):423–34. 10.1016/j.devcel.2015.01.035 25710529 PMC4429600

[19] Evrony GD, Cai X, Lee E, Hills LB, Elhosary PC, Lehmann HS, et al. Single-neuron sequencing analysis of L1 retrotransposition and somatic mutation in the human brain. Cell. 2012;151(3):483–96. 10.1016/j.cell.2012.09.035 23101622 PMC3567441

[20] Poduri A, Evrony GD, Cai X, Walsh CA. Somatic mutation, genomic variation, and neurological disease. Science. 2013;341(6141):1237758. 10.1126/science.1237758 23828942 PMC3909954

[21] Cai X, Evrony GD, Lehmann HS, Elhosary PC, Mehta BK, Poduri A, et al. Single-cell, genome-wide sequencing identifies clonal somatic copy-number variation in the human brain. Cell Rep. 2014;8(5):1280–9. 10.1016/j.celrep.2014.07.043 25159146 PMC4272008

[22] Bedrosian TA, Linker S, Gage FH. Environment-driven somatic mosaicism in brain disorders. Genome Med. 2016;8(1):58. 10.1186/s13073-016-0317-9 27215330 PMC4877941

[23] Muotri AR, Marchetto MC, Coufal NG, Oefner R, Yeo G, Nakashima K, et al. L1 retrotransposition in neurons is modulated by MeCP2. Nature. 2010;468(7322):443–6. 10.1038/nature09544 21085180 PMC3059197

[24] Muotri AR, Zhao C, Marchetto MC, Gage FH. Environmental influence on L1 retrotransposons in the adult hippocampus. Hippocampus. 2009;19(10):1002–7. 10.1002/hipo.20564 19771587 PMC2758700

[25] Faulkner GJ. Retrotransposons: mobile and mutagenic from conception to death. FEBS Lett. 2011;585(11):1589–94. 10.1016/j.febslet.2011.03.061 21477589

[26] De Cecco M, Criscione SW, Peckham EJ, Hillenmeyer S, Hamm EA, Manivannan J, et al. Genomes of replicatively senescent cells undergo global epigenetic changes leading to gene silencing and activation of transposable elements. Aging Cell. 2013;12(2):247–56. 10.1111/acel.12047 23360310 PMC3618682

[27] De Cecco M, Criscione SW, Peterson AL, Neretti N, Sedivy JM, Kreiling JA. Transposable elements become active and mobile in the genomes of aging mammalian somatic tissues. Aging (Albany NY). 2013;5(12):867–83. 10.18632/aging.100621 24323947 PMC3883704

[28] De Cecco M, Ito T, Petrashen AP, Elias AE, Skvir NJ, Criscione SW, et al. L1 drives IFN in senescent cells and promotes age-associated inflammation. Nature. 2019;566(7742):73–8. 10.1038/s41586-018-0784-9 30728521 PMC6519963

[29] Driver CJ, McKechnie SW. Transposable elements as a factor in the aging of Drosophila melanogaster. Ann N Y Acad Sci. 1992;673:83–91. 10.1111/j.1749-6632.1992.tb27439.x 1336649

[30] Elsner D, Meusemann K, Korb J. Longevity and transposon defense, the case of termite reproductives. Proc Natl Acad Sci U S A. 2018;115(21):5504–9. 10.1073/pnas.1804046115 29735660 PMC6003524

[31] St Laurent G 3rd, Hammell N, McCaffrey TA. A LINE-1 component to human aging: do LINE elements exact a longevity cost for evolutionary advantage? Mech Ageing Dev. 2010;131(5):299–305. 10.1016/j.mad.2010.03.008 20346965 PMC2875337

[32] Wood JG, Helfand SL. Chromatin structure and transposable elements in organismal aging. Front Genet. 2013;4:274. 10.3389/fgene.2013.00274 24363663 PMC3849598

[33] Wood JG, Jones BC, Jiang N, Chang C, Hosier S, Wickremesinghe P, et al. Chromatin-modifying genetic interventions suppress age-associated transposable element activation and extend life span in Drosophila. Proc Natl Acad Sci U S A. 2016;113(40):11277–82. 10.1073/pnas.1604621113 27621458 PMC5056045

[34] Maxwell PH, Burhans WC, Curcio MJ. Retrotransposition is associated with genome instability during chronological aging. Proc Natl Acad Sci U S A. 2011;108(51):20376–81. 10.1073/pnas.1100271108 22021441 PMC3251071

[35] Maxwell PH, Curcio MJ. Incorporation of Y’-Ty1 cDNA destabilizes telomeres in Saccharomyces cerevisiae telomerase-negative mutants. Genetics. 2008;179(4):2313–7. 10.1534/genetics.108.089052 18660531 PMC2516100

[36] Maxwell PH, Curcio MJ. Host factors that control long terminal repeat retrotransposons in Saccharomyces cerevisiae: implications for regulation of mammalian retroviruses. Eukaryot Cell. 2007;6(7):1069–80. 10.1128/EC.00092-07 17496126 PMC1951103

[37] Sankowski R, Strohl JJ, Huerta TS, Nasiri E, Mazzarello AN, D’Abramo C, et al. Endogenous retroviruses are associated with hippocampus-based memory impairment. Proc Natl Acad Sci U S A. 2019;116(51):25982–90. 10.1073/pnas.1822164116 31792184 PMC6925997

[38] Criscione SW, Zhang Y, Thompson W, Sedivy JM, Neretti N. Transcriptional landscape of repetitive elements in normal and cancer human cells. BMC Genomics. 2014;15:583. 10.1186/1471-2164-15-583 25012247 PMC4122776

[39] Lee E, Iskow R, Yang L, Gokcumen O, Haseley P, Luquette LJ, 3rd, et al. Landscape of somatic retrotransposition in human cancers. Science. 2012;337(6097):967–71. 10.1126/science.1222077 22745252 PMC3656569

[40] Lock FE, Rebollo R, Miceli-Royer K, Gagnier L, Kuah S, Babaian A, et al. Distinct isoform of FABP7 revealed by screening for retroelement-activated genes in diffuse large B-cell lymphoma. Proc Natl Acad Sci U S A. 2014;111(34):E3534–43. 10.1073/pnas.1405507111 25114248 PMC4151764

[41] Miki Y, Nishisho I, Horii A, Miyoshi Y, Utsunomiya J, Kinzler KW, et al. Disruption of the APC gene by a retrotransposal insertion of L1 sequence in a colon cancer. Cancer Res. 1992;52(3):643–5. 1310068

[42] Rodriguez-Martin C, Cidre F, Fernandez-Teijeiro A, Gomez-Mariano G, de la Vega L, Ramos P, et al. Familial retinoblastoma due to intronic LINE-1 insertion causes aberrant and noncanonical mRNA splicing of the RB1 gene. J Hum Genet. 2016;61(5):463–6. 10.1038/jhg.2015.173 26763876

[43] Scarfo I, Pellegrino E, Mereu E, Kwee I, Agnelli L, Bergaggio E, et al. Identification of a new subclass of ALK-negative ALCL expressing aberrant levels of ERBB4 transcripts. Blood. 2016;127(2):221–32. 10.1182/blood-2014-12-614503 26463425

[44] Scott EC, Gardner EJ, Masood A, Chuang NT, Vertino PM, Devine SE. A hot L1 retrotransposon evades somatic repression and initiates human colorectal cancer. Genome Res. 2016;26(6):745–55. 10.1101/gr.201814.115 27197217 PMC4889970

[45] Teugels E, De Brakeleer S, Goelen G, Lissens W, Sermijn E, De Greve J. De novo Alu element insertions targeted to a sequence common to the BRCA1 and BRCA2 genes. Hum Mutat. 2005;26(3):284. 10.1002/humu.9366 16088935

[46] Wiesner T, Lee W, Obenauf AC, Ran L, Murali R, Zhang QF, et al. Alternative transcription initiation leads to expression of a novel ALK isoform in cancer. Nature. 2015;526(7573):453–7. 10.1038/nature15258 26444240 PMC4807020

[47] Wolff EM, Byun HM, Han HF, Sharma S, Nichols PW, Siegmund KD, et al. Hypomethylation of a LINE-1 promoter activates an alternate transcript of the MET oncogene in bladders with cancer. PLoS Genet. 2010;6(4):e1000917. 10.1371/journal.pgen.1000917 20421991 PMC2858672

[48] Nguyen THM, Carreira PE, Sanchez-Luque FJ, Schauer SN, Fagg AC, Richardson SR, et al. L1 Retrotransposon Heterogeneity in Ovarian Tumor Cell Evolution. Cell Rep. 2018;23(13):3730–40. 10.1016/j.celrep.2018.05.090 29949758

[49] Schauer SN, Carreira PE, Shukla R, Gerhardt DJ, Gerdes P, Sanchez-Luque FJ, et al. L1 retrotransposition is a common feature of mammalian hepatocarcinogenesis. Genome Res. 2018;28(5):639–53. 10.1101/gr.226993.117 29643204 PMC5932605

[50] Shukla R, Upton KR, Munoz-Lopez M, Gerhardt DJ, Fisher ME, Nguyen T, et al. Endogenous retrotransposition activates oncogenic pathways in hepatocellular carcinoma. Cell. 2013;153(1):101–11. 10.1016/j.cell.2013.02.032 23540693 PMC3898742

[51] Rodic N, Sharma R, Sharma R, Zampella J, Dai L, Taylor MS, et al. Long interspersed element-1 protein expression is a hallmark of many human cancers. Am J Pathol. 2014;184(5):1280–6. 10.1016/j.ajpath.2014.01.007 24607009 PMC4005969

[52] Rodic N, Steranka JP, Makohon-Moore A, Moyer A, Shen P, Sharma R, et al. Retrotransposon insertions in the clonal evolution of pancreatic ductal adenocarcinoma. Nat Med. 2015;21(9):1060–4. 10.1038/nm.3919 26259033 PMC4775273

[53] Treger RS, Pope SD, Kong Y, Tokuyama M, Taura M, Iwasaki A. The Lupus Susceptibility Locus Sgp3 Encodes the Suppressor of Endogenous Retrovirus Expression SNERV. Immunity. 2019;50(2):334–47 e9. 10.1016/j.immuni.2018.12.022 30709743 PMC6382577

[54] Wu Z, Mei X, Zhao D, Sun Y, Song J, Pan W, et al. DNA methylation modulates HERV-E expression in CD4+ T cells from systemic lupus erythematosus patients. J Dermatol Sci. 2015;77(2):110–6. 10.1016/j.jdermsci.2014.12.004 25595738

[55] Neidhart M, Rethage J, Kuchen S, Kunzler P, Crowl RM, Billingham ME, et al. Retrotransposable L1 elements expressed in rheumatoid arthritis synovial tissue: association with genomic DNA hypomethylation and influence on gene expression. Arthritis Rheum. 2000;43(12):2634–47. 10.1002/1529-0131(200012)43:12&lt;2634::AID-ANR3&gt;3.0.CO;2-1 11145021

[56] Arru G, Mameli G, Deiana GA, Rassu AL, Piredda R, Sechi E, et al. Humoral immunity response to human endogenous retroviruses K/W differentiates between amyotrophic lateral sclerosis and other neurological diseases. Eur J Neurol. 2018;25(8):1076–e84. 10.1111/ene.13648 29603839

[57] Douville R, Liu J, Rothstein J, Nath A. Identification of active loci of a human endogenous retrovirus in neurons of patients with amyotrophic lateral sclerosis. Ann Neurol. 2011;69(1):141–51. 10.1002/ana.22149 21280084 PMC3052883

[58] Krug L, Chatterjee N, Borges-Monroy R, Hearn S, Liao WW, Morrill K, et al. Retrotransposon activation contributes to neurodegeneration in a Drosophila TDP-43 model of ALS. PLoS Genet. 2017;13(3):e1006635. 10.1371/journal.pgen.1006635 28301478 PMC5354250

[59] Li W, Jin Y, Prazak L, Hammell M, Dubnau J. Transposable elements in TDP-43-mediated neurodegenerative disorders. PLoS One. 2012;7(9):e44099. 10.1371/journal.pone.0044099 22957047 PMC3434193

[60] Li W, Lee MH, Henderson L, Tyagi R, Bachani M, Steiner J, et al. Human endogenous retrovirus-K contributes to motor neuron disease. Sci Transl Med. 2015;7(307):307ra153. 10.1126/scitranslmed.aac8201 26424568 PMC6344353

[61] Tam OH, Rozhkov NV, Shaw R, Kim D, Hubbard I, Fennessey S, et al. Postmortem Cortex Samples Identify Distinct Molecular Subtypes of ALS: Retrotransposon Activation, Oxidative Stress, and Activated Glia. Cell Rep. 2019;29(5):1164–77 e5. 10.1016/j.celrep.2019.09.066 31665631 PMC6866666

[62] Crow YJ, Rehwinkel J. Aicardi-Goutieres syndrome and related phenotypes: linking nucleic acid metabolism with autoimmunity. Hum Mol Genet. 2009;18(R2):R130–6. 10.1093/hmg/ddp293 19808788 PMC2758706

[63] Thomas CA, Tejwani L, Trujillo CA, Negraes PD, Herai RH, Mesci P, et al. Modeling of TREX1-Dependent Autoimmune Disease using Human Stem Cells Highlights L1 Accumulation as a Source of Neuroinflammation. Cell Stem Cell. 2017;21(3):319–31 e8. 10.1016/j.stem.2017.07.009 28803918 PMC5591075

[64] Bollati V, Galimberti D, Pergoli L, Dalla Valle E, Barretta F, Cortini F, et al. DNA methylation in repetitive elements and Alzheimer disease. Brain Behav Immun. 2011;25(6):1078–83. 10.1016/j.bbi.2011.01.017 21296655 PMC3742099

[65] Guo C, Jeong HH, Hsieh YC, Klein HU, Bennett DA, De Jager PL, et al. Tau Activates Transposable Elements in Alzheimer’s Disease. Cell Rep. 2018;23(10):2874–80. 10.1016/j.celrep.2018.05.004 29874575 PMC6181645

[66] Protasova MS, Gusev FE, Grigorenko AP, Kuznetsova IL, Rogaev EI, Andreeva TV. Quantitative Analysis of L1-Retrotransposons in Alzheimer’s Disease and Aging. Biochemistry (Mosc). 2017;82(8):962–71. 10.1134/S0006297917080120 28941465

[67] Sun W, Samimi H, Gamez M, Zare H, Frost B. Pathogenic tau-induced piRNA depletion promotes neuronal death through transposable element dysregulation in neurodegenerative tauopathies. Nat Neurosci. 2018;21(8):1038–48. 10.1038/s41593-018-0194-1 30038280 PMC6095477

[68] Yan Z, Zhou Z, Wu Q, Chen ZB, Koo EH, Zhong S. Presymptomatic Increase of an Extracellular RNA in Blood Plasma Associates with the Development of Alzheimer’s Disease. Curr Biol. 2020;30(10):1771–82 e3. 10.1016/j.cub.2020.02.084 32220323

[69] Perron H, Bernard C, Bertrand JB, Lang AB, Popa I, Sanhadji K, et al. Endogenous retroviral genes, Herpesviruses and gender in Multiple Sclerosis. J Neurol Sci. 2009;286(1–2):65–72. 10.1016/j.jns.2009.04.034 19447411

[70] Perron H, Garson JA, Bedin F, Beseme F, Paranhos-Baccala G, Komurian-Pradel F, et al. Molecular identification of a novel retrovirus repeatedly isolated from patients with multiple sclerosis. The Collaborative Research Group on Multiple Sclerosis. Proc Natl Acad Sci U S A. 1997;94(14):7583–8. 10.1073/pnas.94.14.7583 9207135 PMC23865

[71] Perron H, Suh M, Lalande B, Gratacap B, Laurent A, Stoebner P, et al. Herpes simplex virus ICP0 and ICP4 immediate early proteins strongly enhance expression of a retrovirus harboured by a leptomeningeal cell line from a patient with multiple sclerosis. J Gen Virol. 1993;74 (Pt 1):65–72. 10.1099/0022-1317-74-1-65 7678635

[72] Tan H, Qurashi A, Poidevin M, Nelson DL, Li H, Jin P. Retrotransposon activation contributes to fragile X premutation rCGG-mediated neurodegeneration. Hum Mol Genet. 2012;21(1):57–65. 10.1093/hmg/ddr437 21940752 PMC3235010

[73] Gemenetzi M, Lotery AJ. The role of epigenetics in age-related macular degeneration. Eye (Lond). 2014;28(12):1407–17. 10.1038/eye.2014.225 25233816 PMC4268465

[74] Zhao B, Wu Q, Ye AY, Guo J, Zheng X, Yang X, et al. Somatic LINE-1 retrotransposition in cortical neurons and non-brain tissues of Rett patients and healthy individuals. PLoS Genet. 2019;15(4):e1008043. 10.1371/journal.pgen.1008043 30973874 PMC6478352

[75] Bourque G, Burns KH, Gehring M, Gorbunova V, Seluanov A, Hammell M, et al. Ten things you should know about transposable elements. Genome Biol. 2018;19(1):199. 10.1186/s13059-018-1577-z 30454069 PMC6240941

[76] Wicker T, Sabot F, Hua-Van A, Bennetzen JL, Capy P, Chalhoub B, et al. A unified classification system for eukaryotic transposable elements. Nat Rev Genet. 2007;8(12):973–82. 10.1038/nrg2165 17984973

[77] Boeke JD, Garfinkel DJ, Styles CA, Fink GR. Ty elements transpose through an RNA intermediate. Cell. 1985;40(3):491–500. 10.1016/0092-8674(85)90197-7 2982495

[78] Ashley J, Cordy B, Lucia D, Fradkin LG, Budnik V, Thomson T. Retrovirus-like Gag Protein Arc1 Binds RNA and Traffics across Synaptic Boutons. Cell. 2018;172(1–2):262–74 e11. 10.1016/j.cell.2017.12.022 29328915 PMC5793882

[79] Kawamura Y, Sanchez Calle A, Yamamoto Y, Sato TA, Ochiya T. Extracellular vesicles mediate the horizontal transfer of an active LINE-1 retrotransposon. J Extracell Vesicles. 2019;8(1):1643214. 10.1080/20013078.2019.1643214 31448067 PMC6691892

[80] Pastuzyn ED, Day CE, Kearns RB, Kyrke-Smith M, Taibi AV, McCormick J, et al. The Neuronal Gene Arc Encodes a Repurposed Retrotransposon Gag Protein that Mediates Intercellular RNA Transfer. Cell. 2018;173(1):275. 10.1016/j.cell.2018.03.024 29570995 PMC5923900

[81] Ribet D, Harper F, Dupressoir A, Dewannieux M, Pierron G, Heidmann T. An infectious progenitor for the murine IAP retrotransposon: emergence of an intracellular genetic parasite from an ancient retrovirus. Genome Res. 2008;18(4):597–609. 10.1101/gr.073486.107 18256233 PMC2279247

[82] Burke WD, Malik HS, Rich SM, Eickbush TH. Ancient lineages of non-LTR retrotransposons in the primitive eukaryote, Giardia lamblia. Mol Biol Evol. 2002;19(5):619–30. 10.1093/oxfordjournals.molbev.a004121 11961096

[83] Eickbush DG, Eickbush TH. Vertical transmission of the retrotransposable elements R1 and R2 during the evolution of the Drosophila melanogaster species subgroup. Genetics. 1995;139(2):671–84. 7713424 10.1093/genetics/139.2.671 PMC1206373

[84] Malik HS, Burke WD, Eickbush TH. The age and evolution of non-LTR retrotransposable elements. Mol Biol Evol. 1999;16(6):793–805. 10.1093/oxfordjournals.molbev.a026164 10368957

[85] Malik HS, Henikoff S, Eickbush TH. Poised for contagion: evolutionary origins of the infectious abilities of invertebrate retroviruses. Genome Res. 2000;10(9):1307–18. 10.1101/gr.145000 10984449

[86] Jones BC, Wood JG, Chang C, Tam AD, Franklin MJ, Siegel ER, et al. A somatic piRNA pathway in the Drosophila fat body ensures metabolic homeostasis and normal lifespan. Nat Commun. 2016;7:13856. 10.1038/ncomms13856 28000665 PMC5187580

[87] Sousa-Victor P, Ayyaz A, Hayashi R, Qi Y, Madden DT, Lunyak VV, et al. Piwi Is Required to Limit Exhaustion of Aging Somatic Stem Cells. Cell Rep. 2017;20(11):2527–37. 10.1016/j.celrep.2017.08.059 28903034 PMC5901960

[88] Zhou F, Li M, Wei Y, Lin K, Lu Y, Shen J, et al. Activation of HERV-K Env protein is essential for tumorigenesis and metastasis of breast cancer cells. Oncotarget. 2016;7(51):84093–117. 10.18632/oncotarget.11455 27557521 PMC5356647

[89] Chan SM, Sapir T, Park SS, Rual JF, Contreras-Galindo R, Reiner O, et al. The HERV-K accessory protein Np9 controls viability and migration of teratocarcinoma cells. PLoS One. 2019;14(2):e0212970. 10.1371/journal.pone.0212970 30818388 PMC6394991

[90] Gonzalez-Cao M, Iduma P, Karachaliou N, Santarpia M, Blanco J, Rosell R. Human endogenous retroviruses and cancer. Cancer Biol Med. 2016;13(4):483–8. 10.20892/j.issn.2095-3941.2016.0080 28154780 PMC5250606

[91] Kim A, Terzian C, Santamaria P, Pelisson A, Purd’homme N, Bucheton A. Retroviruses in invertebrates: the gypsy retrotransposon is apparently an infectious retrovirus of Drosophila melanogaster. Proc Natl Acad Sci U S A. 1994;91(4):1285–9. 10.1073/pnas.91.4.1285 8108403 PMC43142

[92] Marlor RL, Parkhurst SM, Corces VG. The Drosophila melanogaster gypsy transposable element encodes putative gene products homologous to retroviral proteins. Mol Cell Biol. 1986;6(4):1129–34. 10.1128/mcb.6.4.1129 3023871 PMC367623

[93] Pelisson A, Song SU, Prud’homme N, Smith PA, Bucheton A, Corces VG. Gypsy transposition correlates with the production of a retroviral envelope-like protein under the tissue-specific control of the Drosophila flamenco gene. EMBO J. 1994;13(18):4401–11. 7925283 10.1002/j.1460-2075.1994.tb06760.x PMC395367

[94] Song SU, Gerasimova T, Kurkulos M, Boeke JD, Corces VG. An env-like protein encoded by a Drosophila retroelement: evidence that gypsy is an infectious retrovirus. Genes Dev. 1994;8(17):2046–57. 10.1101/gad.8.17.2046 7958877

[95] Ribet D, Harper F, Esnault C, Pierron G, Heidmann T. The GLN family of murine endogenous retroviruses contains an element competent for infectious viral particle formation. J Virol. 2008;82(9):4413–9. 10.1128/JVI.02141-07 18287236 PMC2293071

[96] Dewannieux M, Harper F, Richaud A, Letzelter C, Ribet D, Pierron G, et al. Identification of an infectious progenitor for the multiple-copy HERV-K human endogenous retroelements. Genome Res. 2006;16(12):1548–56. 10.1101/gr.5565706 17077319 PMC1665638

[97] Robinson-McCarthy LR, McCarthy KR, Raaben M, Piccinotti S, Nieuwenhuis J, Stubbs SH, et al. Reconstruction of the cell entry pathway of an extinct virus. PLoS Pathog. 2018;14(8):e1007123. 10.1371/journal.ppat.1007123 30080900 PMC6095630

[98] Szymczak AL, Workman CJ, Wang Y, Vignali KM, Dilioglou S, Vanin EF, et al. Correction of multi-gene deficiency in vivo using a single ’self-cleaving’ 2A peptide-based retroviral vector. Nat Biotechnol. 2004;22(5):589–94. 10.1038/nbt957 15064769

[99] Capy P, Langin T, Higuet D, Maurer P, Bazin C. Do the integrases of LTR-retrotransposons and class II element transposases have a common ancestor? Genetica. 1997;100(1–3):63–72. 9440259

[100] Chalvet F, Teysset L, Terzian C, Prud’homme N, Santamaria P, Bucheton A, et al. Proviral amplification of the Gypsy endogenous retrovirus of Drosophila melanogaster involves env-independent invasion of the female germline. EMBO J. 1999;18(9):2659–69. 10.1093/emboj/18.9.2659 10228177 PMC1171345

[101] Syomin BV, Fedorova LI, Surkov SA, Ilyin YV. The endogenous Drosophila melanogaster retrovirus gypsy can propagate in Drosophila hydei cells. Mol Gen Genet. 2001;264(5):588–94. 10.1007/s004380000344 11212913

[102] Brasset E, Taddei AR, Arnaud F, Faye B, Fausto AM, Mazzini M, et al. Viral particles of the endogenous retrovirus ZAM from Drosophila melanogaster use a pre-existing endosome/exosome pathway for transfer to the oocyte. Retrovirology. 2006;3:25. 10.1186/1742-4690-3-25 16684341 PMC1524798

[103] Teysset L, Burns JC, Shike H, Sullivan BL, Bucheton A, Terzian C. A Moloney murine leukemia virus-based retroviral vector pseudotyped by the insect retroviral gypsy envelope can infect Drosophila cells. J Virol. 1998;72(1):853–6. 10.1128/JVI.72.1.853-856.1998 9420299 PMC109448

[104] Lecher P, Bucheton A, Pelisson A. Expression of the Drosophila retrovirus gypsy as ultrastructurally detectable particles in the ovaries of flies carrying a permissive flamenco allele. J Gen Virol. 1997;78 (Pt 9):2379–88. 10.1099/0022-1317-78-9-2379 9292028

[105] Bodea GO, McKelvey EGZ, Faulkner GJ. Retrotransposon-induced mosaicism in the neural genome. Open Biol. 2018;8(7). 10.1098/rsob.180074 30021882 PMC6070720

[106] Upton KR, Gerhardt DJ, Jesuadian JS, Richardson SR, Sanchez-Luque FJ, Bodea GO, et al. Ubiquitous L1 mosaicism in hippocampal neurons. Cell. 2015;161(2):228–39. 10.1016/j.cell.2015.03.026 25860606 PMC4398972

[107] Maxwell PH. What might retrotransposons teach us about aging? Curr Genet. 2016;62(2):277–82. 10.1007/s00294-015-0538-2 26581630 PMC5120397

[108] Savva YA, Jepson JE, Chang YJ, Whitaker R, Jones BC, St Laurent G, et al. RNA editing regulates transposon-mediated heterochromatic gene silencing. Nat Commun. 2013;4:2745. 10.1038/ncomms3745 24201902 PMC3992701

[109] Simon M, Van Meter M, Ablaeva J, Ke Z, Gonzalez RS, Taguchi T, et al. LINE1 Derepression in Aged Wild-Type and SIRT6-Deficient Mice Drives Inflammation. Cell Metab. 2019;29(4):871–85 e5. 10.1016/j.cmet.2019.02.014 30853213 PMC6449196

[110] Blaudin de The FX, Rekaik H, Peze-Heidsieck E, Massiani-Beaudoin O, Joshi RL, Fuchs J, et al. Engrailed homeoprotein blocks degeneration in adult dopaminergic neurons through LINE-1 repression. EMBO J. 2018;37(15). 10.15252/embj.201797374 29941661 PMC6068427

[111] Kaneko H, Dridi S, Tarallo V, Gelfand BD, Fowler BJ, Cho WG, et al. DICER1 deficit induces Alu RNA toxicity in age-related macular degeneration. Nature. 2011;471(7338):325–30. 10.1038/nature09830 21297615 PMC3077055

[112] Tarallo V, Hirano Y, Gelfand BD, Dridi S, Kerur N, Kim Y, et al. DICER1 loss and Alu RNA induce age-related macular degeneration via the NLRP3 inflammasome and MyD88. Cell. 2012;149(4):847–59. 10.1016/j.cell.2012.03.036 22541070 PMC3351582

[113] Carreira PE, Ewing AD, Li G, Schauer SN, Upton KR, Fagg AC, et al. Evidence for L1-associated DNA rearrangements and negligible L1 retrotransposition in glioblastoma multiforme. Mob DNA. 2016;7:21. 10.1186/s13100-016-0076-6 27843499 PMC5105311

[114] Doucet-O’Hare TT, Rodic N, Sharma R, Darbari I, Abril G, Choi JA, et al. LINE-1 expression and retrotransposition in Barrett’s esophagus and esophageal carcinoma. Proc Natl Acad Sci U S A. 2015;112(35):E4894–900. 10.1073/pnas.1502474112 26283398 PMC4568228

[115] Doucet-O’Hare TT, Sharma R, Rodic N, Anders RA, Burns KH, Kazazian HH Jr. Somatically Acquired LINE-1 Insertions in Normal Esophagus Undergo Clonal Expansion in Esophageal Squamous Cell Carcinoma. Hum Mutat. 2016;37(9):942–54. 10.1002/humu.23027 27319353 PMC5548391

[116] Solyom S, Ewing AD, Rahrmann EP, Doucet T, Nelson HH, Burns MB, et al. Extensive somatic L1 retrotransposition in colorectal tumors. Genome Res. 2012;22(12):2328–38. 10.1101/gr.145235.112 22968929 PMC3514663

[117] Tang Z, Steranka JP, Ma S, Grivainis M, Rodic N, Huang CR, et al. Human transposon insertion profiling: Analysis, visualization and identification of somatic LINE-1 insertions in ovarian cancer. Proc Natl Acad Sci U S A. 2017;114(5):E733–E40. 10.1073/pnas.1619797114 28096347 PMC5293032

[118] Wylie A, Jones AE, D’Brot A, Lu WJ, Kurtz P, Moran JV, et al. p53 genes function to restrain mobile elements. Genes Dev. 2016;30(1):64–77. 10.1101/gad.266098.115 26701264 PMC4701979

[119] Yang N, Kazazian HH Jr. L1 retrotransposition is suppressed by endogenously encoded small interfering RNAs in human cultured cells. Nat Struct Mol Biol. 2006;13(9):763–71. 10.1038/nsmb1141 16936727

[120] Carreira PE, Richardson SR, Faulkner GJ. L1 retrotransposons, cancer stem cells and oncogenesis. FEBS J. 2014;281(1):63–73. 10.1111/febs.12601 24286172 PMC4160015

