# Supplementary figures and images for: Intercellular viral spread and intracellular transposition of Drosophila gypsy
Source: PLoS Genet. 2021 Apr 22;17(4):e1009535. doi: 10.1371/journal.pgen.1009535 (PMC8096092; doi:10.1371/journal.pgen.1009535)

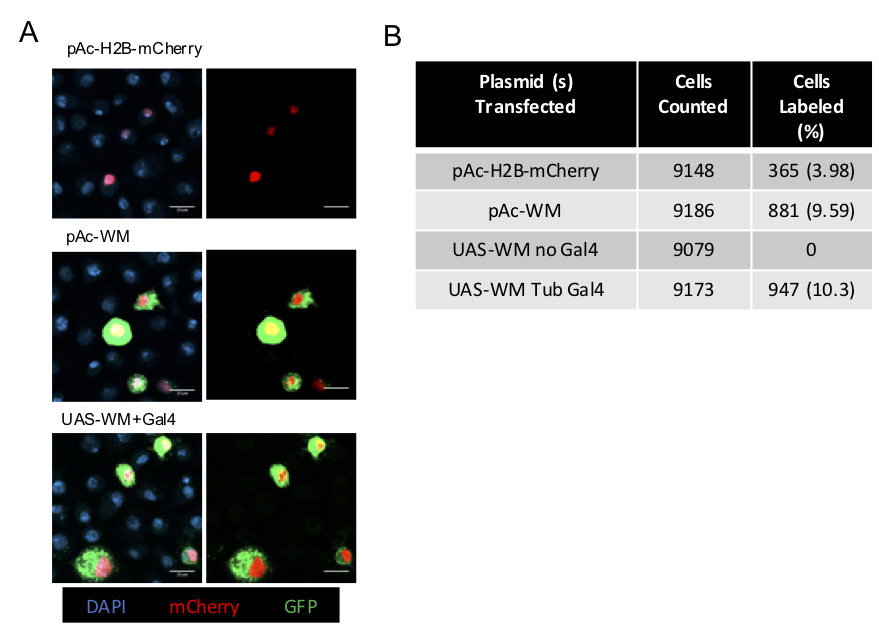

Supplement: S1 Fig — A) Fluorescent images showing mCherry labeled nuclei present in approximately 4% of pAc-H2B-mCherry transfected cells, as well as WM signal expressed in approximately 9.6% and 10.3% of pAc-WM and UAS-WM cotransfected with Tub Gal4 transfected cells respectively. UAS-WM, when not cotransfected with a Gal4 plasmid showed no expression of the WM reporter. B) Quantification of the cells counted and the percentage and number (in parentheses) of cells expressing the control pAc-H2B-mCherry, pAc-WM, and UAS-WM with and without Gal4 plasmids. Quantification is presented as totals cells counted from 3 near equivalent sets of biological replicates. (TIFF) [file pgen.1009535.s001.tiff]

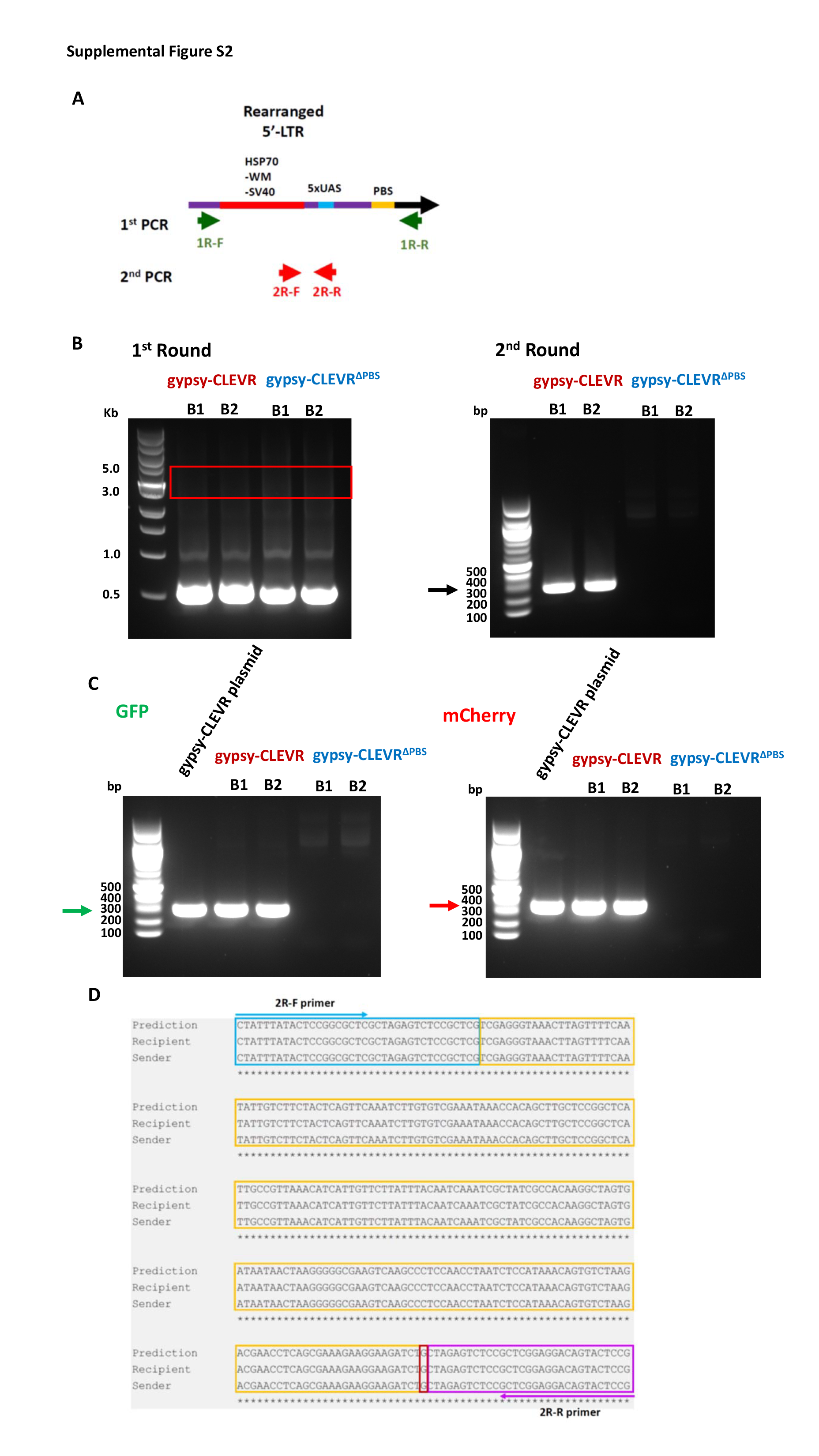

Supplement: S2 Fig — Drosophila S2 cells transfected with Gypsy-CLEVR (sending cells) were grown in a trans-well apparatus opposite to untransfected S2 (recipient) cells. The PBS deletion mutant of this construct was used as a control that can become expressed but cannot be used to generate cDNA. DNA was isolated from recipient cells and gypsy-CLEVR DNA was detected by PCR using several different primer designs. First, a nested PCR scheme (A) was used to selectively amplify gypsy-CLEVR sequences that had undergone the predicted rearrangement that is associated with replication. The first round PCR primers (1R-F and 1R-R) were used to enrich for a 5’ fragment of gypsy elements that included the 5’LTR and internal sequences including the PBS. Products of this reaction (B, left panel) include endogenous gypsy sequences (bright band at ~500nt) as well as a predicted larger fragment derived from the gypsy-CLEVR construct if present. Although not visible on this gel, DNA from the predicted size region (Red rectangle) was isolated and used as template for a second round of PCR using primers that are specific to the HSP70-TATA sequences of the WM reporter (2R-F) and the UAS region (2R-R). This second PCR can only amplify from template that has undergone replication, leading to rearrangement, placing the WM sequence into the 5’-LTR, nearby to the UAS-sequences. This reaction results in amplification of a product of the predicted size (B, right panel) when the wild-type PBS construct is used, but not when using the construct with the PBS mutation. Two independent experiments (Batch 1, B1; Batch 2, B2) yielded similar results. Batch 1 and 2 each consisted of 3 independent trans-well cultures with each construct, and this PCR product was detected in 4 of those 6 experiments with the intact PBS construct and 0 of 6 with the PBS mutant (panel B, right and not shown). We also detect both the GFP and mCherry fluorescent reporters (C) using primers specific to those sequences. Amplification of these pro [file pgen.1009535.s002.tif]

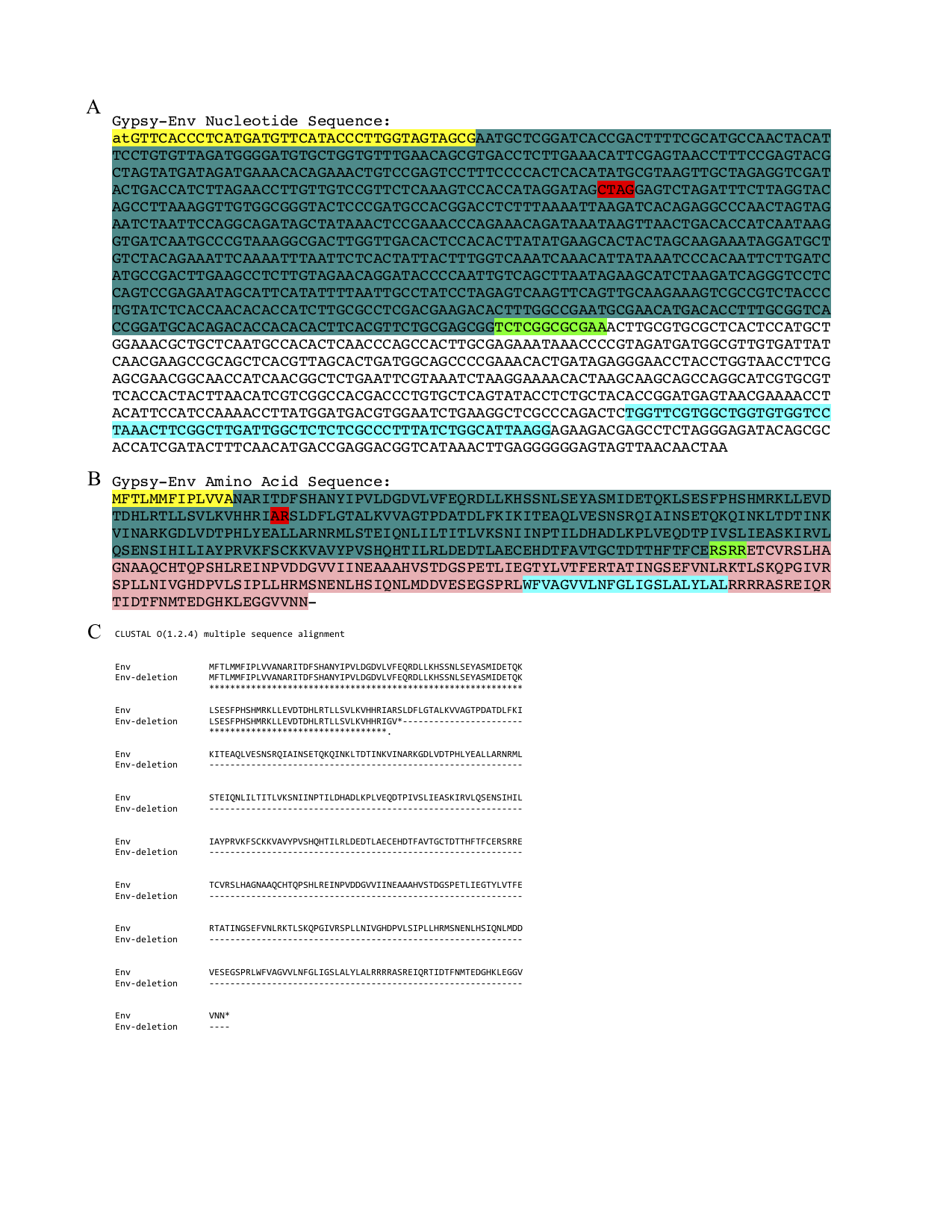

Supplement: S3 Fig — A) Nucleotide sequence of gypsy Env. B) Amino Acid sequence of gypsy Env. C) Clustal analysis comparing WT gypsy Env to gypsy-CLEVREnv_mut.Highlighted portions of the sequence represent the following: Yellow- Signal Peptide, Teal- Surface Protein Domain, Red- Induced Point Mutation, Green- Protein Cleavage Site, Light Blue- Transmembrane Region. (TIFF) [file pgen.1009535.s003.tiff]
